# Construct-level predictive validity of educational attainment and intellectual aptitude tests in medical student selection: meta-regression of six UK longitudinal studies

**DOI:** 10.1186/1741-7015-11-243

**Published:** 2013-11-14

**Authors:** IC McManus, Chris Dewberry, Sandra Nicholson, Jonathan S Dowell, Katherine Woolf, Henry WW Potts

**Affiliations:** 1 UCL Medical School, University College London, Gower Street, London WC1E 6BT, UK; 2 Research Department of Clinical, Educational and Health Psychology, Division of Psychology and Language Sciences, University College London, Gower Street, London WC1E 6BT, UK; 3 Department of Organizational Psychology, Birkbeck, University of London, Malet Street, Bloomsbury, London WC1E 7HX, UK; 4 Institute of Health Science Education, Queen Mary London, Turner Street, London E1 2AD, UK; 5 Undergraduate Medical Education, Ninewells Hospital and Medical School, Dundee, Scotland DD1 9SY, UK

**Keywords:** Medical student selection, Undergraduate performance, Postgraduate performance, Educational attainment, Aptitude tests, Criterion-related construct validity, Range restriction, Right censorship, Grade inflation, Markov Chain Monte Carlo algorithm

## Abstract

**Background:**

Measures used for medical student selection should predict future performance during training. A problem for any selection study is that predictor-outcome correlations are known only in those who have been selected, whereas selectors need to know how measures would predict in the entire pool of applicants. That problem of interpretation can be solved by calculating construct-level predictive validity, an estimate of true predictor-outcome correlation across the range of applicant abilities.

**Methods:**

Construct-level predictive validities were calculated in six cohort studies of medical student selection and training (student entry, 1972 to 2009) for a range of predictors, including A-levels, General Certificates of Secondary Education (GCSEs)/O-levels, and aptitude tests (AH5 and UK Clinical Aptitude Test (UKCAT)). Outcomes included undergraduate basic medical science and finals assessments, as well as postgraduate measures of Membership of the Royal Colleges of Physicians of the United Kingdom (MRCP(UK)) performance and entry in the Specialist Register. Construct-level predictive validity was calculated with the method of Hunter, Schmidt and Le (2006), adapted to correct for right-censorship of examination results due to grade inflation.

**Results:**

Meta-regression analyzed 57 separate predictor-outcome correlations (POCs) and construct-level predictive validities (CLPVs). Mean CLPVs are substantially higher (.450) than mean POCs (.171). Mean CLPVs for first-year examinations, were high for A-levels (.809; CI: .501 to .935), and lower for GCSEs/O-levels (.332; CI: .024 to .583) and UKCAT (mean = .245; CI: .207 to .276). A-levels had higher CLPVs for all undergraduate and postgraduate assessments than did GCSEs/O-levels and intellectual aptitude tests. CLPVs of educational attainment measures decline somewhat during training, but continue to predict postgraduate performance. Intellectual aptitude tests have lower CLPVs than A-levels or GCSEs/O-levels.

**Conclusions:**

Educational attainment has strong CLPVs for undergraduate and postgraduate performance, accounting for perhaps 65% of true variance in first year performance. Such CLPVs justify the use of educational attainment measure in selection, but also raise a key theoretical question concerning the remaining 35% of variance (and measurement error, range restriction and right-censorship have been taken into account). Just as in astrophysics, ‘dark matter’ and ‘dark energy’ are posited to balance various theoretical equations, so medical student selection must also have its ‘dark variance’, whose nature is not yet properly characterized, but explains a third of the variation in performance during training. Some variance probably relates to factors which are unpredictable at selection, such as illness or other life events, but some is probably also associated with factors such as personality, motivation or study skills.

## Background

Selection of medical students in the UK and elsewhere depends heavily on prior measures of educational attainment, which in the UK mainly consists of GCE A-levels, AS-levels and General Certificates of Secondary Education (GCSEs), and Scottish Qualifications Authority (SQA) Highers and Advanced Highers. Such measures are currently problematic, in part because of continuing grade inflation, resulting in more and more students getting maximum grades, and partly because of concerns that educational attainment may reflect differences in secondary school quality, with the diversity of applicants and entrants thereby being reduced. As a result, in the past decade or so many medical schools in the UK, Australia, New Zealand and elsewhere have used additional selection measures such as tests of intellectual aptitude, examples being the UK Clinical Aptitude Test (UKCAT), Biomedical Admissions Test (BMAT), Undergraduate Medicine and Health Sciences Admission Test (UMAT) and Graduate Medical School Admissions Test (GAMSAT) [1].

The use of both educational attainment and intellectual ability for selection has been questioned because of doubts about how well they predict undergraduate performance at medical school (predictive validity) [1,2]. A more general concern is that postgraduate performance, when doctors are in practice, should be predicted. Few studies have related postgraduate outcomes to educational attainment at secondary school, although the few that do suggest there are significant correlations [3,4], resulting in what we have called the Academic Backbone, achievement at each academic stage, before, during and after medical school, predicting subsequent performance in assessments [4]. In the present paper, we assess the predictive validity and the construct-level predictive validity of measures of educational attainment and intellectual ability, for undergraduate and postgraduate measures of achievement, in six prospective studies in the UK of medical school selection. In particular, we assess the theoretically crucial issue of the strength of the construct-level predictive validity of educational attainment and intellectual ability in medical student selection.

Construct-level predictive validity is a complex concept with a complex history [5-7], although in principle it is straightforward, at least in the statistically defined way in which we wish to use it, which follows the usage of Hunter *et al.*[8]. The construct-level predictive validity of a selection measure in the context of medical school performance refers to the association between the construct assessed by the selection measure, the predictor and the medical knowledge, skills and attitudes measured by later undergraduate and postgraduate examinations, the outcomes. No measure is perfect, and construct-level predictive validity takes that into account. Rather than simply specifying the correlation between scores on a measure of medical knowledge and scores on a measure used during selection to predict the capacity to acquire that knowledge, construct-level predictive validity estimates the correlation between the underlying trait, knowledge or skill measured by the selection test, and the underlying medical knowledge measured in the examinations. If it were the case that, say, educational attainment were a perfect predictor of subsequently acquiring medical knowledge, then construct-level predictive validity, the “true predictor-outcome correlation”, would be exactly one. In practice, no predictor could assess such an outcome perfectly, in part because predictors and outcomes are measured unreliably, and hence any actual correlation would fall short of unity. The calculation of construct-level predictive validity takes unreliability and other practical problems of measuring the predictor-outcome into account, and hence estimates true predictor-outcome correlation, the correlation which would be found between the underlying construct measured by the outcome and the underlying construct measured by the predictor in an ideal world with ideal measures.

A deep problem for assessing selection is that while selection takes place in the entire pool of candidates or applicants, validation of the predictor measures can only take place in those who have entered medical school. However, the students admitted necessarily have higher and less variable scores on the predictor than those who are rejected, because those predictor scores are used as an integral part of the selection process. Predictor scores in those selected also have a smaller range (standard deviation) than in applicants overall. Restriction of range inevitably reduces the actual or empirical correlation which can be found between predictors and outcomes, meaning that actual predictor-outcome correlations in entrants to medical school are necessarily much smaller than the “true predictor-outcome correlations”, the construct-level predictive validity coefficients. The principles underlying the estimation of construct-level predictive validity, particularly in the presence of restriction of range, unreliability and right-censorship are discussed in the section below.

### Restriction of range, unreliability, right-censorship and construct-level predictive validity

**Figure 1:**
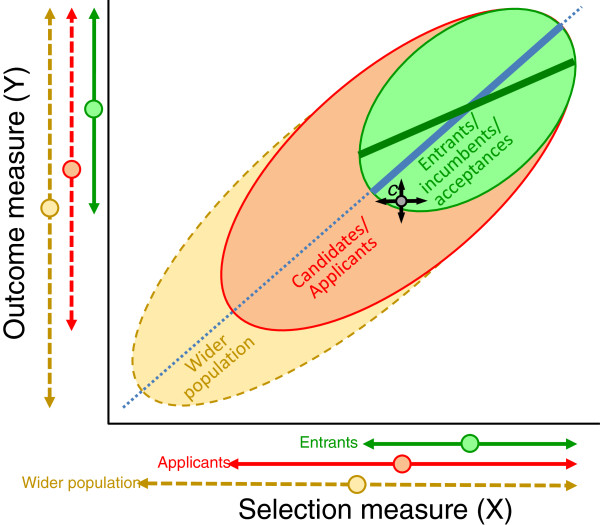
**Restriction of range in medical school applicants and entrants.** See text within *Restriction of range, unreliability, right-censorship and construct-level predictive validity* section for further details.

The statistical theory behind construct-level predictive validity can be understood intuitively by thinking about the process of selection as a whole, as is shown diagrammatically in Figure 1. From a selector’s point of view, a group of candidates or applicants apply for a course, a job or a post. They are shown in red in Figure 1. If a valid selection measure is available then selectors assess that measure in all of the applicants, and they have a range of scores, shown schematically by the red arrow and circle at the bottom of the figure, to indicate the mean and the range or standard deviation. Selectors then use scores on the selection measure to determine which applicants are to be accepted, the group of entrants, incumbents or acceptances. Selection may depend entirely on the selection measure (direct selection) or it can depend on the selection measure and other information about applicants (indirect selection). As Hunter *et al*. [8] have shown, most selection is indirect. Entrants are shown in green in Figure 1, and the arrows at the bottom show they have a higher average score than applicants, and, of particular importance, their range or standard deviation is lower. Although selectors typically have little knowledge or control over the process, another stage of selection occurred earlier in which applicants self-selected themselves from a wider population of individuals who might have applied but did not in fact do so or did not even consider doing so. The wider population is by the orange-brown lines in Figure 1, and they probably have lower selection scores and a wider range than actual applicants, self-knowledge of their likely selection scores in part explaining the reason for not applying. The wider population is shown as dashed lines as less accurate information is available for them. Scores on the selection measure are available for the entrants, the applicants and, sometimes, the wider population.

To be effective in selection, a measure has to be a valid predictor of the outcome measure, which is shown on the vertical axis, and is usually job or course performance. The dotted, blue diagonal line in Figure 1 shows the relationship of the outcome measure to the selection measure. The relationship is not, of course, perfect and, hence, the data are scattered in an ellipse around the line, with the ratio of the short axis to the long axis being proportional to the correlation. The more tightly the points are clustered around the line, then the higher the correlation. Correlations depend in part on the range or variance in the x and y measures (and in the extreme case where all of the x values are the same, there is necessarily a correlation of zero). The effect of the range can be seen in Figure 1, where the green ellipse for the entrants has a lower correlation than that in the candidates, who in turn have a lower correlation than does the orange ellipse for the wider population.

The fundamental statistical problem in assessing selection measures is that the correlation between the outcome measure and the selection measure is only known in those who have been accepted (that is, the green ellipse in Figure 1, the relationship there being shown by the solid blue line). However, the correlation in entrants is inevitably lower than the correlation in applicants because of restriction of range. The validity of a selection measure is not indicated by how well it differentiates between those who have already been selected (which is rarely a useful thing to know in practical terms), but by predicting how badly candidates with lower selection scores would have performed on the course were they to have been admitted. The correlation between the selection and outcome measures is known as the construct-level predictive validity of the selection measure. By making some reasonable assumptions about underlying processes, the construct-level predictive validity can be inferred from the correlation of selection and outcome measures in entrants, and then applied to all applicants rather than just those who are selected.

So far, an assumption has been made that selection and outcome measures are measured without error, that is, if a person had their scores measured on two separate occasions then those two scores would be identical. In practice, that never happens, and any behavioural measure shows measurement error. In Figure 1 the gray circle shows the true selection and outcome scores for a candidate, *c*, with the arrows indicating the likely errors in that measurement. If *c* is a weak candidate then their true score may have happened to be below that required for selection, but they got lucky; and likewise strong candidates can occasionally have error against them and they are not selected. Without measurement error, the relationship between the selection measure and the outcome measure would be the blue solid and dotted lines of Figure 1. Measurement error, though, results in the fitted line (the regression line), having a lower slope than the true line (and that is indicated by the solid green line in the group of entrants, in whom that relationship is measured). As an additional complication, estimates of the reliability of the selection measure will be lower if calculated only in the group of entrants, because of restriction of range.

**Figure 2:**
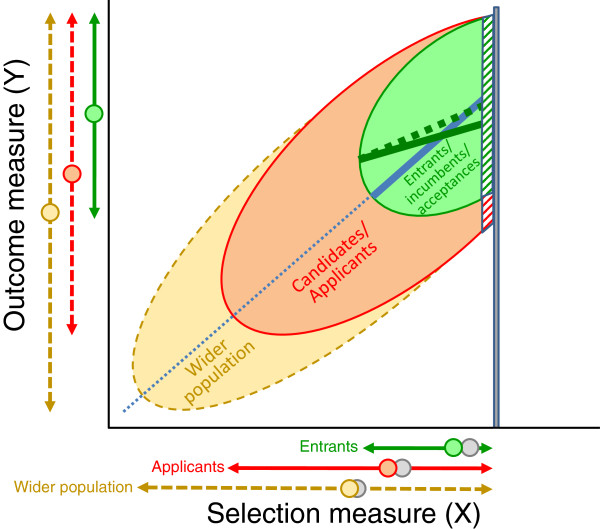
**The effect of right-censorship on restriction of range in medical school applicants and entrants.** See text within *Restriction of range, unreliability, right-censorship and construct* section for further details.

Finally, an additional problem for medical student selection is shown in Figure 2, where the selection measure is right-censored due to a ceiling effect. Candidates who would have had high selection measures are restricted in the scores they can attain. The result is that the actual correlation of selection and outcomes measures in the entrants, shown by the solid green line is less steep (a lower correlation), than it would have been without right-censorship (shown by the dashed green line in Figure 2).

### The importance of construct-level predictive validity

A key error in selection is to assess the validity of selection measures by looking at correlations in those who have entered medical school, such correlations often seeming to be disappointingly small, to the extent that even in prestigious journals a naïve interpretation can be made that selection measures, such as A-level grades, are actually of little value [9]. Within medicine, four decades ago, in 1973, Sir George Smart made exactly the same error when he said at a UK’s General Medical Council (GMC) conference that,

“As predictors of future performance [,] examinations were not highly successful, as was shown by the low correlation of A level GCE grades with subsequent performance in medical school” [10] (p. 5).

However thirty years before that, in 1943, Burt was already talking of the “time-honoured fallacy”, of,

“judging the efficiency of [an] examination as a mean of *selection* by stating its efficiency as a means of predicting the order of merit *within* the selected group” [11] (p. 2).

The fallacy, rightly so-called and very prevalent, is that correlations within a selected group are useful indicators of the true predictive validity of a selection measure. In fact, they are measuring something of little real interest, which is the ability of a test to predict how students who enter medical school will actually perform in medical school. What selectors really need to know is how well all applicants, not only those selected but also those rejected, would have performed in medical school were they to have been accepted. Construct-level predictive validity provides an estimate of precisely that. The fallacy is easily seen in a simple thought experiment. Imagine that all accepted students gain AAA at A-level. Although the correlation with medical school performance would necessarily be zero, that would not mean that an applicant admitted with grades of EEE would also perform equally well.

If construct-level predictive validity, the “true predictor-outcome correlation”, is known, then it has great theoretical importance. Were construct-level predictive validity to be one, then in principle the predictor and the outcome measure equivalent, parallel processes, and the predictor is indeed valid. It may not be perfect in practice, but that is something that can be improved upon by test refinement to improve reliability, and so on. If, however, the construct-level predictive validity is less than one then there is a strong theoretical implication that even though the predictor may be measuring something useful, something else must also be important in predicting the remaining variance in the outcome. And whatever that something else is, it must necessarily be conceptually distinct from and statistically independent of the predictor measure. In the case of medical student selection it may be personality, motivation, communicative ability, life events or whatever, which are not measured by selection tests. The important thing is that a construct-level predictive validity of less than one for a predictor, such as educational attainment, sets limits on the capacity of that particular predictor to explain outcomes, and other predictors must therefore also be sought. A very practical implication of such a theoretical analysis of construct-level predictive validity is that it emphasizes where efforts in selection can and should be made. Were prior educational attainment to have a construct-level predictive validity of one then it, and it alone, should be the focus of selection, assuming that the major concern of selectors is that future students and doctors should be able to acquire adequate clinical knowledge and hence pass examinations (and students who fail examinations and leave medical school certainly do not go on to become doctors). Were, however, educational attainment’s construct-level predictive validity to be less than one then selection should search for and take into account those other characteristics which in part contribute to whether or not students and doctors are better able to pass examinations.

The statistical challenge of estimating construct-level predictive validity is to work backwards from the “actual predictor-outcome correlation” to the “true predictor-outcome correlation”. The principles of that process have been known for many decades [5,11-15], and the problem is now, in general, statistically tractable [8,16]. As well as the actual predictor-outcome correlation, such methods of calculation require information on the distribution of predictor scores in both entrants and medical school applicants, and reliability estimates are also needed, both for the predictor variable in the pool of applicants, and the outcome variable in the entrants. Given those, construct-level predictive validity can be estimated, using the method of Hunter *et al*. [8]. In the present case there are also two other technical issues. First, as we show in the statistical appendix (Additional file 1), the Hunter *et al.* method is effective if all of the measures are normally distributed, but it can produce erroneous results if the predictor measure is heavily ‘right-censored’, as is the case for A-levels and Highers, where many candidates have maximum scores of 3 As at A-level or 5 As at Highers. Second, the Hunter *et al.* method does not provide estimates of the standard error or the confidence intervals of estimates of construct-level predictive validity. The solution for both problems, which we have implemented, is to modify the Hunter *et al.* method for right-censored distributions (and also for binary or ordinal outcome measures, as occurs in some cases), using the Markov chain Monte Carlo (MCMC) algorithm (see later). It is then possible to estimate construct-level predictive validities with standard errors of the estimates. The details of the method are shown in the statistical appendix (Additional file 1).

### Attainment vs aptitude

Selection measures used in medicine can be broadly divided into measures of attainment or achievement and measures of aptitude or ability [1]. Attainment tests, such as GCSEs and A-levels in the UK, typically assess knowledge and skills acquired during formal education, high achievement probably requiring not only intellectual ability but also motivation, appropriate study skills, and personality traits, such as conscientiousness and openness to experience. MCAT, used for selecting medical students in the United States [17], is clearly a measure of substantive understanding of basic sciences and is also an attainment test. In contrast, aptitude or ability tests, such as UKCAT and BMAT in the UK, emphasize, “intellectual capabilities for thinking and reasoning, particularly logical and analytical reasoning abilities” [18], and are regarded as measures of potential, independent of educational opportunity, and in many ways are conceptually similar to general mental ability or intelligence.

Implicit in the use of measures of academic attainment and of aptitude is an assumption that the measures assess skills or abilities which underpin performance in the undergraduate medical course and in postgraduate training and professional achievement. The major difference between selection based on aptitude and on attainment is that selection based on aptitude tests assumes that generic or specific thinking and reasoning skills are important predictors of medical school performance, whereas for attainment tests it is assumed that the substantive content of subjects, such as of biology or chemistry, is of direct help in subsequent medical training, and/or that attaining such basic scientific knowledge is an indirect indicator of motivation, intellectual ability or personality [2].

### The present study

In the present study our primary aim is to assess the predictive and construct-level validity of measures of secondary school attainment in the UK in predicting performance not only in undergraduate medical school examinations, but also in postgraduate training, where we will consider the Membership of the Royal Colleges of Physicians of the United Kingdom (MRCP(UK)), a major postgraduate medical examination taken by many UK medical graduates, as well as entry into the General Medical Council’s (GMC’s) Specialist Register. In addition, we also consider data on the predictive validity of aptitude tests, considering both the AH5 [19], an intelligence test specifically designed for university students, and the UKCAT [20], a test currently used in a majority of UK medical schools, data on the predictive validity of which have been presented in the UKCAT-12 study of 12 UK medical schools [21].

Small-scale studies of selection have little statistical power for estimating construct-level predictive validity and, therefore, in the present study we will estimate construct-level predictive validity in six large-scale cohort studies which have taken place in the UK over the past three and a half decades using a range of predictor and outcome measures. We have used meta-regression [22] to assess how construct-level predictive validities differ in relation to the outcome measures assessed (Basic Medical Sciences, Finals, MRCP(UK) and Specialist Register), to type of predictor measure (A-level, AS-level, GCSE, Higher, Advanced Higher, and intellectual aptitude tests (UKCAT and AH5)), and the year in which students entered medical school (1972 to 2009).

### Overview of the datasets

The data for the present study come from six cohort studies analyzed in detail elsewhere, so only a summary is provided here. In order of year of entry of the students, the Westminster Study [3] is the oldest (entry 1972 to 1980), followed by the 1980 [23], 1985 [24] and 1990 [25] Cohort Studies (entry in 1981, 1986 and 1991), the University College London Medical School (UCLMS) Cohorts (entry 2001 to 2004) [26] and the UKCAT-12 Study [21] (entry 2007 to 2009). Four of the studies, the 1980, 1985 and 1990 Cohort Studies and UKCAT-12, are proper selection studies in that data are available not only for entrants to medical school but also for applicants. The remaining two studies, the Westminster Cohort and the UCLMS Cohorts have data only on entrants but the four selection studies proper allow estimates of the distributions of applicant measures in those two studies. The Westminster Cohort has a full-length timed intellectual aptitude test (AH5), the 1990 Cohort has an abbreviated AH5, and UKCAT-12 administered the UKCAT. Follow-up through the years of medical school is most detailed in the UCLMS Cohorts, and the UKCAT-12 data analyzed here only include first year performance. UKCAT-12 is, though, the largest study followed by the 1990 Cohort, all cohorts, except for UCLMS, have data on which doctors are on the Specialist Register, and the 1990 and UCLMS Cohorts have MRCP(UK) results.

## Method

Six separate cohort studies were analyzed. Summaries of the studies are provided below, and more details are available elsewhere [4,21]. In reverse order of medical school entry, the studies were:

### The UKCAT-12 study

Twelve UK medical schools (four in Scotland) that used UKCAT as a part of their selection took part in this study. Overall 1,666, 1,768 and 1,442 students entered the 12 medical schools in 2007, 2008 and 2009. Undergraduate performance was available as an overall score for the end of the first year of the course, and within each year of entry and medical school was expressed as a z-score (mean = 0, SD = 1) to allow comparability across the medical schools and cohorts. UKCAT scores were analyzed as the total score (range 1,200 to 3,600). Educational achievement was expressed as the total score on three best A-levels (scored A = 10, B = 8, C = 6, D = 4 and E = 2), four best AS-levels (scored as A-levels), nine best GCSEs (scored as A* = 6, A = 5, B = 4, C = 3, D = 2 and E = 1), five best SQA Highers (scored as A = 10, B = 8, C = 6 and D = 4), five best SQA “Highers plus” (scored as A1 = 10, A2 = 9, B3 = 8, B4 = 7, C5 = 6, C6 = 5, D7 = 4 and D8 = 3), and single best SQA Advanced Highers (scored as Highers Plus). Previous analyses [27] had also shown that the various measures of previous examination attainment could be combined into a single measure. For GCE examinations, the scores for the three best A-levels, four best AS-levels, nine best GCSEs, as well as grades in A-level Biology, Chemistry, Math, Physics and General Studies were combined, using EM (Expectation-Maximization) imputation to replace missing values, and then extraction of the first principal component. A similar process took place for SQA qualifications, combining the five highest Highers Plus grades, highest Advanced Highers grade, Highers Plus grade at Biology, Chemistry, Physics and Math, and Advanced Higher grade at Biology, Chemistry, Math and Physics, with EM imputation for missing values and extraction of the first principle component. We refer to these measures here as “EducationalAttainmentGCE” and “EducationalAttainmentSQA”.

### The UCLMS cohort study

The sampling frame for this study [4] consisted of 729 students entering the clinical course (year 3) at University College London Medical School (UCLMS) in autumn 2005 (n = 383) and 2006 (n = 346), of whom 621 (85.2%) had studied basic medical sciences (BMS) at UCLMS, and all but one of the remaining 108 students had studied BMS at Oxford or Cambridge.

Students had entered medical school between 2001 and 2004, different times since entry reflecting personal circumstances, exam failure or intercalated degrees. Finals were mostly taken in 2007 and 2008, with some students taking them later, again for various reasons. Examination results were available for students taking first and second year exams at UCL, and for all third, fourth and fifth year examinations. Performance was summarized by the medical school as a total overall score. Because students entered the medical school in different years, comparability was ensured by converting all scores to z-scores by year.

A-levels were taken by 669 students and scored as the best three grades attained, on the basis of A = 10, B = 8, C = 6, D = 4 and E = 2 (A* grades had not yet been introduced). A total of 62.5% of students achieved the maximum of 30 points, with 16.9%, 12.3%, 3.1%, 2.2% and 2.9% achieving 28, 26,24, 22 or 20 (or fewer) points. GCSE results were known for 599 students, students taking an average number of 10.04 GCSEs and achieving a mean of 53.6 points (SD 7.84; A* = 6, A = 5, B = 4, C = 3, D = 2, E = 1).

Of the original 729 students, 252 (34.6%) had taken MRCP(UK) Part 1 by October 2012, 122 (16.7%) had taken Part 2, and 59 (8.1%) had taken PACES, with Parts 1, 2 and PACES passed by 80.9%, 90.2% and 76.3%. Performance was obtained from the records of MRCP(UK) Central Office, based on a ‘History file’ extracted on 12 October 2012. For Part 1 and Part 2, marks are expressed as percentage points above or below the pass mark (which varies from diet to diet). For PACES/nPACES, marks were expressed as a percentage relative to the pass mark, as in a previous study [28]. All MRCP(UK) marks are analyzed in relationship to the mark at the first attempt, which has been shown to be a good indicator of overall performance [29]. None of the cohort was on the specialist register at the time of follow-up.

### The 1990 cohort study

The sampling frame was the 6,901 applicants to English medical schools in the autumn of 1990 for admission in 1991 (St. Mary’s Hospital Medical School; UMDS (United Medical and Dental Schools of Guy’s and St. Thomas’s); UCMSM (University College and Middlesex School of Medicine), University of Sheffield, and University of Newcastle-upon-Tyne) [25]. Applicants who entered any UK medical school have been followed up, in their final medical school year (mostly in 1996 or 1997 [30,31], in their pre-registration house officer (PRHO) year (mostly in 1997 or 1998 [32,33]), in 2002, when the doctors were mostly working as GPs or Specialist Registrars [34], and again in 2009 [35]. UK medical schools provided information on preclinical/basic medical science course outcomes in 1993 to 1994 and on finals in 1996 to 1997 to ascertain the outcome in clinical years. Basic medical science performance was expressed on a four-point ordinal scale, and finals performance on a binary scale.

A-level results were scored in the standard way. The study took place as O-levels were being replaced with GCSEs, and separate scores were derived for mean O-level grade or mean GCSE grade, and expressed as z-scores. Applicants who attended for interview at St. Mary’s, UMDS or Sheffield took an abbreviated version of the AH5 test of intelligence [19] (aAH5), which was timed. The aAH5 was entirely for research purposes, and results were not made available to the medical schools concerned.

GMC numbers for all graduates were identified, and subsequently used to link the data with the GMC’s LRMP (List of Registered Medical Practitioners), and with MRCP(UK) results, which were scored in a similar way to that in the UCLMS Cohorts with minor differences [4].

### The 1985 cohort study

The 1985 cohort study [24] consisted of 2,399 individuals who applied to St. Mary’s Hospital Medical School in the autumn of 1985 for entry to medical school in October 1986. St. Mary’s was a popular choice with applicants with 24.7% of all medical school applicants, including it as one of their five medical school applications. Entrants to any UK medical school were followed up, and included 22.7% of all entrants to UK medical schools in that year [24]. A-level and O-level results of candidates were recorded. UK medical schools provided information on performance on the basic medical science course, recorded on a four-point scale. For students taking finals in the (then) constituent medical schools of the University of London, which had a common, shared examination system, details of performance in all assessments were collected and expressed as a single overall score [36]. Information on MRCP(UK) was not available, but there was information about which doctors were in the GMC’s Specialist Register.

### The 1980 cohort study

The 1980 Cohort Study, which was the first and hence smallest of the three cohort studies at St. Mary’s Hospital Medical School, studied all 1,361 individuals who in the autumn of 1980 applied to study medicine at St. Mary’s. The 519 entrants to any UK medical school were followed up [23,37,38], and represented 12.9% of all UK medical school entrants in 1981. UK medical schools provided information on basic medical science performance on a four-point scale [39]. For students taking the common finals examinations of the University of London, detailed performance measures were available, as with the 1985 cohort study [36].

### The Westminster cohort study

The Westminster Study was initiated by Dr Peter Fleming, who studied the 511 students entering the clinical course of the Westminster Medical School between 1975 and 1982 [3]. The Westminster only ran a clinical course, and basic medical sciences had been studied elsewhere, so that students entered medical training between 1972 and 1980. Outcome on the clinical course was recorded on a four-point scale. A-level results were available for the entrants, and all students also took a timed version of the full AH5 test. Information on which doctors were on the specialist register was available.

### Statistical analysis

Conventional statistical analyses used SPSS 20.0. (International Business Machines Corporation, Statistical Package for the Social Sciences, Armonk, New York, USA) Special purpose programs were written in Matlab to calculate correlations corrected for right-censoring, as well as tetrachoric and polychoric correlations for grouped data. In addition the Hunter-Schmidt-Le (HSL) model of construct-level predictive validity extended for censored and grouped data was also programmed in Matlab. All Matlab programs used the DRAM adaptation of MCMC [40], available from Dr Marko Laine of the University of Helsinki (see helios.fmi.fi/~lainema/mcmc/, helios.fmi.fi/~lainema/mcmc/mcmcstat.zip and helios.fmi.fi/~lainema/dram/). MCMC analyses typically used a chain length of 5,000 or 10,000 with parameter estimates based on the final 2,000 items in the chain, means and standard deviations being used as the estimate and the standard error of parameters, with 5% confidence intervals estimated as the 2.5^th^ and 97.5^th^ percentiles of the actual values in the chain.

The MCMC program used to estimate construct-level predictive validity estimated seven parameters (mean and SD of the predictor in entrants, mean and SD of the predictor in applicants, mean and SD of the outcome measure in entrants, and the correlation between the predictor and the outcome), in each case taking into account right-censorship of measures (for continuous measures such as A-levels), or non-normality and reduced numbers of ordinal outcomes, as for some outcome measures (such as four-point summaries of BMS performance). The correlation and the SD estimates of the predictor in applicants and entrants, as well as the two reliabilities, were then entered into the HSL formula [8]. The MCMC algorithm typically had a chain length of 5,000, with estimates derived for the last 2,000 iterations. Estimates were plotted against chain number to ensure that equilibrium had been reached. The HSL formula was calculated separately for each step in the chain, and hence standard errors could be calculated for the construct-level predictive validity, selection ratio and other parameters.

Meta-regression of the construct-level predictive validities was carried out using the *Moderator_r* macro (*Meta_Mod_r.sps*) for *SPSS* of Field and Gillett [41]. All analyses used random effects regression analysis, and hence are generalizable to other populations than those used in the present analyses.

All confidence intervals (CI) are 95% confidence intervals, whatever the method of calculation.

### Ethics

The Chair of the UCL Ethics Committee has confirmed that studies, such as the present ones, are exempt from needing formal permission from the Committee, being included under sections *c* and *f* of the exemptions (see http://ethics.grad.ucl.ac.uk/exemptions.php).

## Results

**Figure 3:**
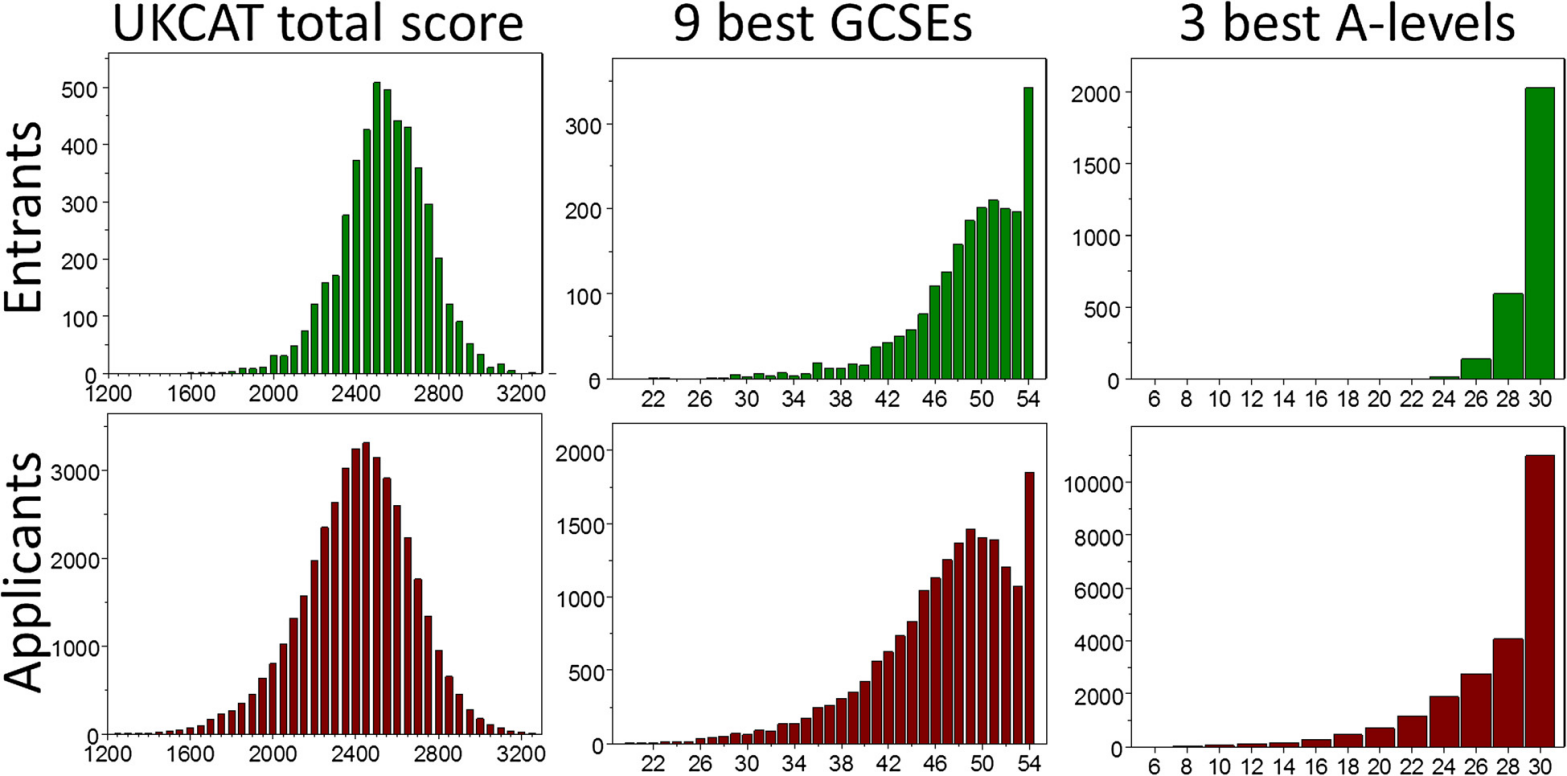
**Distributions of UKCAT and GCE examination results.** Distributions in the UK Clinical Aptitude Test (UKCAT)-12 study of total UKCAT scores, the nine best General Certificates of Secondary Education (GCSEs) and the three best A-levels in Entrants (top) and Applicants (bottom).

**Figure 4:**
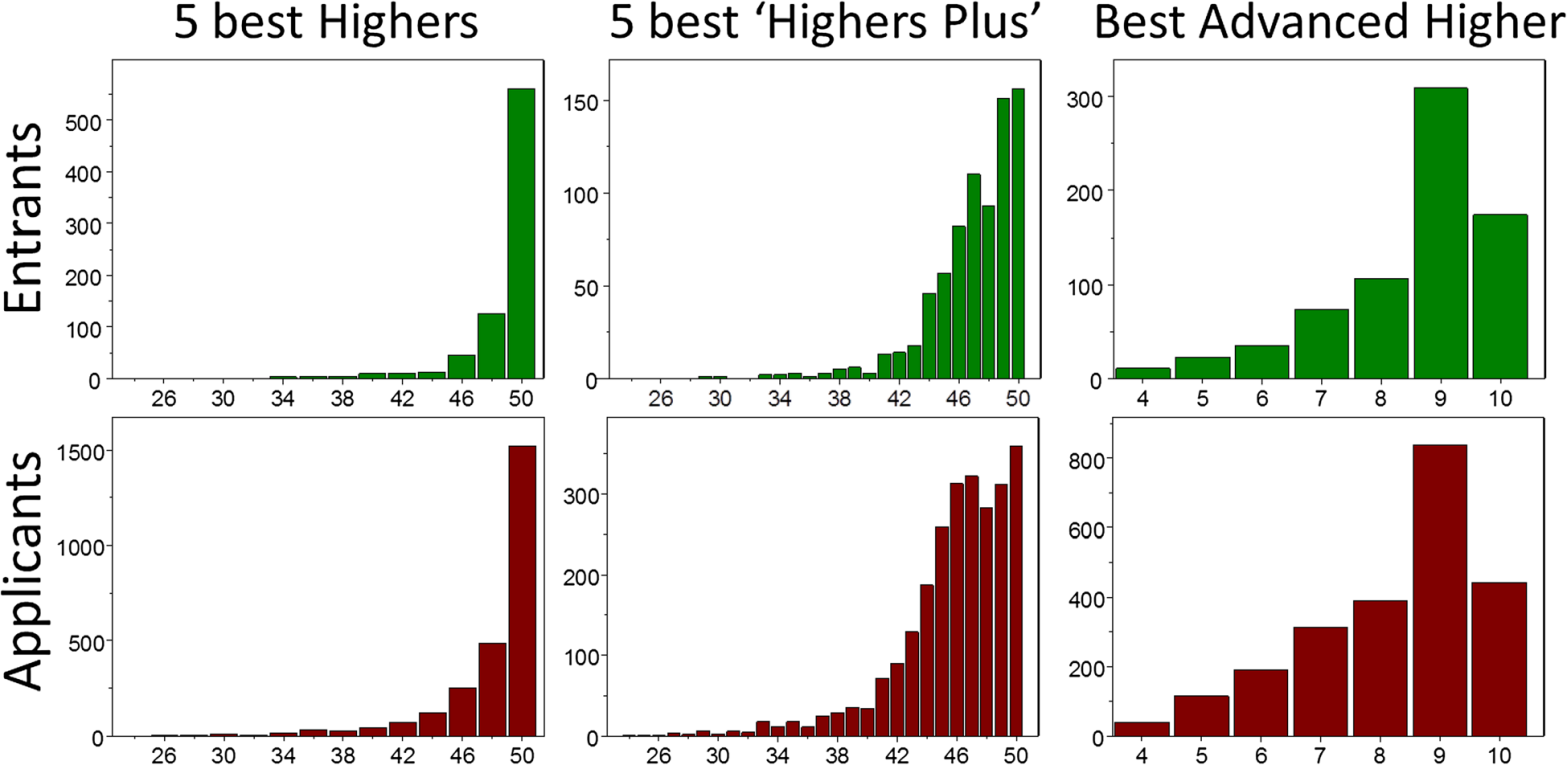
**Distribution of SCE examination results.** Distributions, in the UKCAT-12 study, of five best Highers, five best ‘Highers Plus’ (see text), and the best Advanced Higher in Entrants (top) and Applicants (bottom). SCE, specialty certificate examination.

The analysis of construct-level predictive validity requires information on the distribution of predictors not only in entrants but also applicants. Data are shown for the UKCAT-12 Study, since it is the largest and most recent study. Figure 3 shows, for the UKCAT-12 study for the years 2007 to 2009, the distribution in entrants and applicants of their three best A-levels (Ns = 277 and 22,744), nine best GCSEs (Ns = 2,104 and 18,494), and UKCAT total score (Ns = 4,811 and 40,401), and Figure 4 shows similar results for SQA Highers (Ns = 773 and 2,582), ‘Highers Plus’ (Ns = 767 and 2,539), and SQA Advanced Highers (Ns = 732 and 2,326). As expected, distributions in entrants are shifted to the right compared with distributions in applicants. The distribution for UKCAT is approximately normal, with the others right-censored. The distribution for GCSEs shows the right-censored normal distribution particularly well. Results for earlier cohorts for A-levels and GCSEs/O-levels are similar but shifted more to the left and were less right-censored [4].

Predictive validity, in the simple unadjusted sense, was calculated separately for each outcome measure and each predictor measure in each of the cohorts, as the Pearson correlation between predictor and outcome, uncorrected for right-censorship, range restriction or attenuation due to lack of reliability; these correlations are, therefore, typical of the calculations which could be carried out by an admissions tutor in a medical school. Table 1 summarizes the 57 predictor-outcome correlations, broken down by Predictor, Outcome and Cohort. The mean sample size is 935, and the mean unweighted correlation .171, and an overall effect in a random effects meta-analysis of .171 (CI: .147 to .195). The effect size is therefore small, and for *P* < .05, with 90% power of finding a significant effect in a one-tailed test, a sample size of 290 would be needed, meaning that only very large medical schools would be likely to find a significant effect when looking at a single year of applicants. Even for the largest simple correlation, between A-levels and first-year BMS results, where the weighted mean correlation is .211 (CI: .144 to .275), a sample of 189 would be required.

**Table 1:** Descriptive statistics for predictor-outcome correlations and criterion-related construct validities

			**Predictor-outcome correlations**		**Criterion-related construct validities**	
		**N participants**	**Pearson**	Corrected*	**r**	**Equivalent N**
	**N studies**	**Mean (Range)**	**Mean (Range)**	**Mean (Range)**	**Mean (Range)**	**Mean (Range)**
**Predictor**						
A-levels	22	**920** (R 55; 3,096)	**.180** (R .041; .306 )	**.231** (R .053; .415)	**.634** (R .147; .943)	**61** (R 8; 245)
AS-levels	1	**1911**	**.182**	**.228**	**.458**	**266**
GCSEs/O-levels	20	**849** (R 52; 2,657)	**.162** (R -.026 ; .269)	**.176** (R −.045; .273)	.**336** (R −.079; .626)	**231** (R 16; 1,271)
Highers	1	**777**	**-.001**	**.076**	**.107**	**336**
‘HighersPlus’	1	**771**	**.143**	**.180**	**.292**	**265**
Advanced Highers	1	**735**	**.345**	**.358**	**.506**	**247**
Ed. Attainment GCE	1	**2768**	**.312**	**.347**	**.923**	**745**
Ed. Attainment SQA	1	**722**	**.419**	**.425**	**.623**	**317**
Aptitude tests (AH5, UKCAT)	9	**934** (R 156; 4,841)	**.132** (R .037; .276)	**.152** (R .045; .260)	**.228** (R.062; .449)	**445** (R 62; 2,720)
**Outcome**						
BMS first year	15	**1521** (R 542; 4,841)	**.207** (R −.001 ; .419)	**.248** (R .076; .425)	**.498** (R .107; .943)	**517** (R 24; 2,720)
BMS overall	9	**1152** (R 502; 3,096)	**.174** (R .037; .282)	**.215** (R .053; .394)	**.491** (R .065; .903)	**203** (R 10; 513)
Finals	11	**786** (R 314; 2,413)	**.187** (R .051 ; .306)	**.222** (R .080; .328)	**.488** (R.097; .871)	**136** (R 10; 389)
MRCP(UK) Pt1	5	**492** (R 202; 957)	**.192** (R .126; .245)	**.221** (R .143; .308)	**.456** (R .168; .692)	**107** (R 32; 209)
MRCP(UK) Pt2	5	**363** (R 98; 753)	**.205** (R .085 ; .299)	**.227** (R .071; .217)	**.442** (R .326; .743)	**44** (R 12; 80)
MRCP(UK) Clinical	5	**277** (R 52; 597)	**.141** (R .058; .236)	**.144** (R .071; .217)	**.317** (R .147; .627)	**40** (R 10; 81)
On Specialist Register	9	**984** (R 393; 2,664)	**.084** (R -.026; .419)	**.113** (R −.045; .249)	**.367** (R −.079; .803)	**126** (R 8; 2,720)
**Cohort**						
Westminster	4	**470** (R 454; 486)	**.169** (R .146 ; .190)	**.226** (R .188; .249)	**.565** (R .386; .803)	**49** (R 17; 96)
1980 cohort	8	**449** (R 314; 562)	**.164** (R −.026; .306)	**.192** (R −.046; .315)	**.457** (R −.079; .864)	**73** (R 14; 178)
1985 cohort	6	**643** (R 347; 851)	**.164** (R .066 ; .240)	**.187** (R .102; .253)	**.566** (R .214; .903)	**98** (R 8; 289)
1990 cohort	18	**1234** (R 156; 3,096)	**.133** (R .037; .276)	**.157** (R .045; .280)	**.357** (R .062; .692)	**190** (R 42; 513)
UCLMS cohorts	12	**362** (R 52; 668)	**.218** (R .058; .299)	**.261** (R .071; .415)	**.475** (R .147; .743)	**96** (R 10; 346)
UKCAT-12	9	**1,938** (R 722; 4841)	**.198** (R −.001; .419)	**.232** (R .076; .425)	**.467** (R .107; .943)	**688** (R 25; 2720)
*All construct validities*	*57*	***935*** *(SD = 956) (R 52; 4841)*	***.171*** *(SD = .092) (R .−026; .419)*	***.203*** *(SD = .101) (*R *−.045; .425)*	**.** ***450*** *(SD = .248) (*R *−.079; .943)*	***213*** *(SD = 396) (*R 8; *2720)*

*The corrected correlation takes into account both right-censoring and the use of ordinal values (see statistical appendix for details).

Note that figures in brackets are ranges (indicated by R:) and are not confidence intervals.

Calculation of construct-level predictive validity is more complex than that of calculating predictor-outcome correlations. The basic method of Hunter *et al.* for indirect range restriction requires the estimation of five parameters: i) the reliability of the predictor measure in applicants; ii) the reliability of the outcome measure in entrants; iii) the predictor-outcome correlation in entrants; iv) the standard deviation of the predictor measure in applicants; and v) the standard deviation of the predictor measure in entrants.

The standard deviations in applicants and entrants are used to calculate the ‘selection ratio’, the SD of the predictor in entrants as a proportion of the SD of the predictor in all applicants, smaller values indicating a greater extent of selection. A selection ratio of one means that entrants have the same variability as applicants (and so, in effect, little or no selection is taking place on the predictor). The mean selection ratio is .732, meaning that entrants indeed have a smaller range of scores than do applicants. The selection ratios differ, however, for different predictors. There is strong selection on the GCE qualifications of A-levels (.656), AS-levels (.667), and GCSEs/O-levels (.676), with less strong selection on the SQA qualifications of Highers (.896), ‘Highers Plus’ (.814), and Advanced Highers (.941). The two derived measures from the UKCAT-12 study [21], Educational Attainment based on GCE results and SQA results, have stronger selection than their component measure (GCE .358; SQA .766), with particularly strong selection on GCEs. The implication is that admissions tutors are making holistic judgments which implicitly combine a wide range of information from different sources. The selection ratio for aptitude tests is only well assessed in the UKCAT-12 study, where the ratio is .775, indicating fairly strong selection, although not as strong as for A-levels.

Selection ratios were not available for the Westminster Cohort or the UCLMS cohorts. Modeling suggested that selection ratios differed little by cohort or by outcome variable, but did show some variation according to the predictor variable. Median values of .664, .690 and .750 were used for A-levels, GCSEs/O-levels and aptitude tests in these cohorts.

Estimation of reliabilities was not always straightforward, particularly for measures such as the three best A-levels, the standard measure of A-level achievement. Estimates of the reliability of A-levels, AS-levels, GCSEs, Highers, Highers Plus and Advanced Highers are generally not available [42]. The calculation of reliabilities from raw data, which is not simple, is described in the statistical appendix (Additional file 1), taking right-censorship into account in each case. The reliability of UKCAT is published in its annual reports [20,43,44], and the reliability of AH5 was based on the values described in the manual. Estimates of outcome measures are also not straightforward. A meta-analysis of grade-point averages finds a reliability of about .84 [45], and that, along with other data, forms the basis for our estimates described in the statistical appendix (Additional file 1). A special problem in some cases is that outcome measures have only three or four ordinal categories (for example, Fail, Re-sit, Pass, Honors), or in the case of being on the Specialist Register are binary. Methods equivalent to tetrachoric and polychoric correlations are described in the statistical appendix (Additional file 1). Estimates of the reliability of MRCP(UK) Parts 1 and 2 have been published [46,47], although they are based on all candidates, rather than UK graduates, and have, therefore, been corrected. A reliability estimate for MRCP(UK) Part 2 Clinical Examination (PACES) is also available [48].

The reliabilities of the various predictors and outcomes are summarized in the Additional file 1: Table S1 and S3. Reliabilities sometimes need to be corrected for right-censorship. Taken overall the predictors had an average reliability of .815, and the outcome measures had a mean reliability of about .834. Reliabilities were not available for all measures, in which case estimates were used (see the statistical appendix (Additional file 1) for details).

### Meta-regression

In total, 57 construct-level predictive validity coefficients and their associated confidence intervals were available, based on a variety of summative outcome measures. Descriptive statistics are given in Table 1 for the simple (Pearson) predictor-outcome correlations, the corrected predictor-outcome correlations, and the construct-level predictive validities, broken down in each case by predictor, outcome and cohort. Construct-level predictive validity coefficients, which take into account reliability, range restriction and right-censorship, are substantially larger (mean = .450) than are the corrected correlations (mean = .203), which in turn are larger than the simple, unadjusted predictor-outcome correlations (mean = .171). All of the participants are used for calculating construct-level predictive validities, as in calculating the simple predictor-outcome correlations (that is, the mean number of participants is 935). However, although construct-level predictive validities are, in effect, correlations, their standard errors cannot be calculated on the basis of the actual N in a study. Instead, standard errors of the construct-level predictive validities were estimated from the variability in the chain of the MCMC algorithm (see statistical appendix (Additional file 1)). The construct-level predictive validities are correlations and, hence, can be entered into a meta-regression. However, meta-regression normally requires r and a value of N to calculate the standard error of correlations before combining them. Since the standard errors of the construct-level predictive validities have been estimated in our case by the MCMC algorithm, we have used those standard error estimates to back-calculate, using the standard formula for the standard error of a correlation, what the “equivalent N” would have been to have resulted in the actual standard error which the MCMC algorithm found. The equivalent N, which is entered into the meta-regression along with the construct-level predictive validity, is shown in Table 1 and it is always smaller than the actual N, showing how construct-level predictive validities are estimated much less reliably than conventional correlations. ‘Equivalent N’ has a mean of 218, and so, on average, equivalent N is about one quarter of actual N, meaning that the standard errors are about twice as large as that expected based on actual N, the difference arising because construct-level predictive validities incorporate uncertainty from several different sources.

The meta-regression analysis of construct-level predictive validity began with a series of exploratory analyses. A categorical effects model with all of the Predictors, Outcomes and Cohorts which has 8 + 6 + 5 = 19 parameters (which is large compared to the 57 data points), found highly significant differences between Predictors (chi-square = 114.4, 8 df, *P* < .001), but not between Outcomes (chi-square = 4.66, 6df, *P* = .588) or Cohorts (chi-square = 5.30, 5 df, *P* = .380). In order to reduce the number of parameters, Cohort and Outcome were expressed as continuous variables (that is, single degrees of freedom), in terms of YearOfEntry to medical school (1975, 1981, 1986, 1991, 2002 and 2008 for the Westminster, 80, 85 and 90 cohorts, UCLMS and UKCAT-12 cohorts), and YearOfTraining (BMS1 = 1, BMSoverall = 2; Finals = 5; MRCP(UK) Parts 1, 2 and Clinical = 8, 9 and 10 , and Specialist Register = 12). A model with Year of Entry and Year of Training as covariates, and Predictor as a categorical measure found significant effects for Predictor (chi-square = 126.0, 8 df, *P* < .001), the effect of YearOfTraining was almost significant (b = −.016, t = −1.91, 45 df, *P* = .063), and would have been significant with a one-tailed test, the effect being in the obvious direction (*P* = .032). The effect of YearOfEntry was not significant (b = −.003, t = −.737, 45 df, *P* = .465). Addition of a term for a YearOfTraining x YearOfEntry interaction also was not significant (t = −.599, 44 df, *P* = .553). Construct-level predictive validity differs therefore between different predictors, and perhaps between Outcomes (outcomes earlier in training having higher validities than later outcomes), but there was no evidence for a YearOfEntry (Cohort) effect, or for a YearOfEntry × YearOfTraining interaction.

The next analyses consider A-levels, GCSEs/O-levels and aptitude tests separately. Table 2 summarizes the meta-analytically combined construct-level predictive validities for the three predictors with reasonable numbers of estimates (A-levels, GCSEs/O-levels and Aptitude Tests), and the various outcome measures, which are also grouped into all BMS (year 1 and 2 measures), all undergraduate measures, all postgraduate (all MRCP and postgraduate measures) and all outcome measures.

**Table 2:** Summary of construct validity coefficients

**Outcome**	**Predictor**		
	**A-levels**	**GCSEs/O-levels**	**Aptitude tests**
First year BMS	**.809** n = 3 (CI .501; .935)	**.332** n = 3 (CI .024; .583)	**.245** n = 1 (CI .207; .276)
BMS overall	**.744** n = 4 (CI .518; .872)	**.361** n = 4 (CI .305; .413)	**.065** n = 1 (CI −.049; .180)
All BMS	**.772** n = 7 (CI .627; .865)	**.338** n = 7 (CI .205; .459)	**.164** n = 2 (CI −.031; .347)
Finals	**.625** n = 5 (CI .449; .754)	**.400** n = 4 (CI .274; .513)	**.226** n = 2 (CI −.093; .503)
*All Undergraduate except first year BMS*	***.684*** *n = 9 (CI .561; .778)*	***.379*** *(n = 8) (CI .316; .439)*	***.147*** *(n = 3) (−.065; .346* ***)***
*All Undergraduate*	***.723*** *n = 12 (CI .616; .803)*	***.359*** *n = 11 (CI .255; .455)*	***.181*** *n = 4 (CI .055; .302)*
MRCP(UK) Part 1 (written)	**.661** n = 2 (CI .523; .765)	**.433** n = 2 (CI .098; .680)	**.168** n = 1 (CI .044; .308)
MRCP(UK) Part 2 (written)	**.502** n = 2 (CI −.030; .812)	**.372** n = 2 (CI .153; .555)	**.358** n = 1 (CI .174; .559)
MRCP(UK) Clinical	**.303** n = 2 (CI .010; .547)	**.498** n = 2 (CI .068; .772)	**.226** n = 1 (CI .007; .422)
*All MRCP*	**.506** n = 6 (CI .301; .666)	**.447** n = 6 (CI .300; .573)	**.226** n = 3 (CI .108; .339)
Specialist Register	**.627** n = 4 (CI .450; .756)	**.119** n = 3 (CI −.044; .276)	**.258** n = 2 (CI -.171; .605)
*All postgraduate*	***.556*** *n = 10 (CI .426; .663)*	***.316*** *n = 9 (CI .148; .466)*	***.243*** *n = 5 (CI .090; .385)*
***All undergraduate and postgraduate***	***.656*** *n = 22 (CI .574; .726)*	***.342*** *n = 20 (CI .258; .420)*	***.208*** *n = 9 (CI .124; .289)*

Construct validities are combined meta-analytically as Fisher’s Z-transforms and then back-transformed to the conventional correlation scale. The number of construct validities and the 95% confidence interval (CI) for the construct validities are also shown. Where n = 1 the confidence intervals are those for the single estimate.

#### Construct-level predictive validity of A-levels

There were 22 construct-level predictive validities for A-levels. Overall A-levels had a construct-level predictive validity which was significantly different from zero (mean = .656; CI .572 to .727). There was no evidence of a YearOfEntry effect or of a YearOfEntry × YearOfTraining interaction, but the YearOfTraining effect was significant (b = −.040, t = −2.267, 19 df, *P* = .035), with no evidence of additional differences between Outcomes after YearOfTraining was taken into account. Table 2 shows that the construct-level predictive validity of A-levels is greatest for first year BMS exams, and declines through undergraduate and postgraduate years, although it is significant in all cases.

#### Construct-level predictive validity of GCSEs/O-levels

Twenty construct-level predictive validities were available for GCSEs/O-levels, with the overall construct-level predictive validity being highly significant (mean = .342; CI .258 to .420). YearOfTraining showed no significant effect on its own (t = −.834, 17 df, p = .416) as neither did YearOfEntry (t = .002, 17 df, *P* = .738). Finally, although neither Linear YearOfEntry and Linear YearOfTraining was significant when both were in the model, when combined with the linear × linear interaction, while YearOfEntry was not significant (*P* = .166), but YearOfTraining was just significant (b = −5.62, t = −2.14, 15 df, *P* = .049), and the interaction was also just (*P* = .049). Taken together there is a suggestion that construct-level predictive validity of GCSEs/O-levels might decline a little as training progresses and in more recent years, but the effects are unclear.

#### Construct-level predictive validity of aptitude tests

Nine construct-level predictive validities were available for aptitude tests, two from the Westminster Cohort (AH5), six from the 1990 Cohort (aAH5), and one from UKCAT-12 (UKCAT total score), with a highly significant effect overall (mean = .208; CI .113 to .299, t = 4.89, 9df, *P* < .00001). Assessed separately, YearOfEntry and YearOfTraining had no effect (*P* = .300 and *P* = .565), although once again when YearOfEntry, YearOfTraining and their interaction were included there were almost significant effects of YearOfTraining (*P* = .081) and the interaction (*P* = .081).

#### Construct-level predictive validity of A-levels, GCSEs/O-levels and Aptitude tests for Undergraduate performance

A-levels, GCSEs/O-levels and Aptitude tests all show significant construct-level predictive validities overall. Here we compare their construct-level predictive validities for the 27 assessments in the undergraduate course, be it basic medical sciences or clinical assessments. The three predictors are significantly different in their construct-level predictive validity (Chi-square = 40.92, 2df, *P* < .001), and as can be seen in Table 2, the construct-level predictive validity for A-levels is .723 (CI: .616 to .803), that for GCSEs/O-levels is .359 (CI: .255 to .455) and .181 (CI: .055 to .302) for aptitude tests.

#### Construct-level predictive validity of A-levels, GCSEs/O-levels and Aptitude tests for Postgraduate performance

Construct-level predictive validity was available for 24 postgraduate outcomes. A-levels, GCSEs/O-levels and aptitude tests showed highly significant differences (chi-square = 9.57, 2df, *P* = .008), and Table 2 shows that A-levels had the highest construct-level predictive validity (mean = .556; CI: .426 to .663), followed by GCSEs/O-levels (mean = .316; CI: .148 to .466) and aptitude tests (mean = .243; CI: .090 to .385). Pairwise comparison showed that A-levels had higher construct-level predictive validity than GCSEs/O-levels and Aptitude Tests (chi-square = 5.535 and 11.14, 1 df, *P* = .019 and < .001), but GCSEs/O-levels were not significantly different from Aptitude Tests (chi-square = .321, 1 df, *P* = .571).

#### Prediction of MRCP(UK) vs Specialist Register

Postgraduate performance was assessed by two rather different outcomes, performance on MRCP(UK) and entry to the Specialist Register. As we have discussed in the paper on the Academic Backbone [4], entry to the Specialist Register is potentially a different form of outcome measure to MRCP(UK) which consists of examination results. We have therefore carried out an analysis comparing the 15 validities based on MRCP(UK) results with 9 validities based on entry to the Specialist Register, across all Predictors (AH5, n = 5; A-levels, n = 10; and GCSEs/O-levels, n = 9). Although there were clear differences in construct validities between the different predictors (chi-square = 10.09, 2df, *P* = .006), there were no significant differences between outcomes coded as MRCP(UK) or Specialist Register (chi-square = 1.003, 1df, *P* = .317). It can be concluded that although MRCP(UK) and Specialist Register may be different conceptually, they are predicted in equivalent ways to one another by earlier measures of secondary school attainment and aptitude.

#### Comparing prediction of undergraduate and postgraduate performance

**Figure 5:**
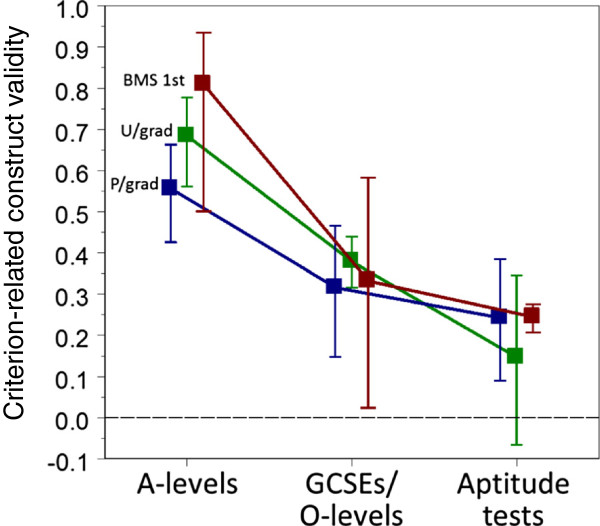
**Criterion-related construct validity.** Meta-analytic estimates with 95% confidence intervals of criterion-related construct validity for A-levels, General Certificates of Secondary Education (GCSEs)/O-levels and aptitude tests, separately for first-year Basic Medical Sciences (BMS) (red; n = 3, 3, 1), all other undergraduate assessments (green; n = 9, 8, 3)) and postgraduate assessments (blue; n = 10, 9, 5).

For undergraduate examinations, the construct-level predictive validities of A-levels, GCSEs/O-levels and Aptitude tests were significantly different, but that was not the case for GCSEs/O-levels and aptitude tests for postgraduate performances (see Figure 5). Considering all 51 construct-level predictive validities, a model with dummy variables for A-levels, GCSEs/O-levels, Aptitude tests and UG/PG was explored in various combinations. Although A-levels always had higher validity than other predictors, the most parsimonious model included just a dummy variable for A-levels, which was highly significant (t = 7.26, 48 df, *P* < .001). After including A-levels, no other variable when added in on its own was significant, although GCSEs/O-levels approached significance (*P* = .098), as did a dummy variable for postgraduate exams (*P* = .116). No interaction terms were significant. Overall, it can be concluded that A-levels are better predictors than GCSEs overall, which are perhaps better predictors than aptitude tests in undergraduates (although the interaction with UG/PG is not significant). Although overall the validities were slightly higher in undergraduate assessments (mean = .485; CI: .406 to .557) than in postgraduate assessments (mean = .386; CI: .282 to .481), that effect did not quite reach significance either on its own (*P* = .104) or after taking A-levels into account (*P* = .116).

#### Construct-level predictive validity of A-levels, GCSEs/O-levels and aptitude tests for first year Basic Medical Science performance

Predicting first-year performance is particularly important, as although a number of students fail and leave medical school then, those who only just get into the second year, with marks little above those who have failed, tend to continue on to the end of the course, and into practice, often struggling for much of the time [49-51]. As a result, construct-level predictive validities were analyzed for just those assessments. The meta-regression contained three relevant construct-level predictive validities for A-levels, .709 (.467 to .880) in the 1980 cohort, .672 (.550 to .775) in the UCLMS cohorts, and .943 (.890 to .980) in UKCAT-12, the latter being by far the largest study. The meta-analytic combined estimate for A-levels is .809 (n = 3; CI: .490 to .937), with no evidence of heterogeneity (chi-square = 2.184, 2 df, *P* = .335). The combined estimate for GCSEs/O-levels was .332 (n = 3; CI: .024 to .583). There was only one construct-level predictive validity of an aptitude test for first year results, in the UKCAT-12 cohort, it being .245 (CI: .207 to .276).

#### AS-levels, Highers, Advanced Highers and educational attainment measures

SQA qualifications were only available for the UKCAT-12 study, and hence their construct-level predictive validities are best compared with those for A-levels, AS-levels and GCSEs in UKCAT-12, which were .943 (CI: .890 to .980), .458 (CI: .359 to .449) and .110 (CI: .058 to .167). Highers, ‘Highers Plus’ and Advanced Highers had construct-level predictive validities of .107 (CI: -.010 to .202), .293 (CI: .189 to .409) and .507 (CI: .429 to .614), none of which compared with that for A-levels, and only Advanced Highers was comparable with AS-levels. In the UKCAT-12 study, two derived measures were also extracted, which we called EducationalAttainmentGCE and EducationalAttainmentSQA, and which were composites derived from all of the educational qualifications. The construct-level predictive validity for EducationalAttainmentGCE was also high at .923 (CI: .912 to .933) and that for EducationalAttainmentSQA was higher than its component parts at .623 (CI: .541 to .676).

## Discussion

Any measure, be it physical, biological or behavioral, has errors due to unreliability. The measures used in medical student selection also suffer from range restriction, and in addition, as Figures 3 and 4 show, many of the educational measures show right-censorship, typically due to grade inflation, with many candidates being at the ceiling. In consequence, selection measures such as A-level grades often seem to show very small correlations with outcome measures, which typically assess medical school examination performance. A typical predictor-outcome correlation in the present study is .171, with the implication that only studies with nearly 300 students would have a 90% chance of finding a significant correlation between a typical predictor and a typical outcome. Such small correlations, particularly if non-significant, are often erroneously treated as meaning that selection variables are ineffective or of no consequence.

Actual predictor-outcome correlations are often far smaller than construct-level predictive validities (true-score correlations). That difference matters because, as Hunter and Schmidt [52] have emphasized, “what we are interested in scientifically is the construct-level correlation” (p.16). Rubin [53] has emphasized that “we really care about the underlying scientific process that is generating [the] outcomes that we happen to see - that we, as fallible researchers, are trying to glimpse through the opaque window of imperfect empirical studies” [53] (p.157).

In a perfect world there would be perfect measures of academic performance at medical school and perfect measures of educational attainment and intellectual aptitude in applicants applying to medical school and entrants to medical school would be a random sample of those applying. Given that, it would be straightforward to determine how well selection measures work, and whether the measures in use are sufficient or perhaps others, assessing other characteristics or traits, are also needed.

Construct-level predictive validities estimate the correlations that would pertain in a world permitting perfectly accurate and complete measurement, and in so doing make several things possible. First, predictors can be compared with one another without reliabilities and range restriction confounding the differences. Second, construct-level predictive validities also provide a perspective on the limits of what current measures could, in principle, do if they were not subject to measurement error or other problems. That is central to the difficult question of whether current measures should be refined, replaced or supplemented by other measures. Finally, because they attempt to consider perfect measures, construct-level predictive validities also throw into sharp relief the theoretical imperfection of even the best measures that we might have, showing their flaws and their conceptual failings. The end result is an assessment of what the measures can in principle do.

### Comparing predictors

Comparing the main predictors, particularly for undergraduate examinations, it is clear that A-levels are the best predictor (.723; CI: .616 to .803), followed by GCSEs/O-levels (.359; CI: .255 to .455), with intellectual aptitude tests predicting much less well, albeit significantly differently than zero (.181; CI: .055 to .302). Other predictors are mostly present only in the UKCAT-12 study and, hence, it is more difficult to generalize about them. However, it does appear that SQA qualifications have a lower construct-level predictive validity than GCE qualifications, with Highers having a very low validity. The lower construct-level predictive validity of SQA qualifications is important because a simple comparison of predictor-outcome correlations suggests that SQA examinations perform better than GCE examinations [21,27]. That the construct-level predictive validities are the other way around is a result of SQAs having higher reliabilities and higher selection ratios (see Table 1), which results in relatively lower construct-level predictive validities^a^. The two composite measures of EducationalAttainmentGCE and EducationalAttainmentSQA, despite having higher correlations with medical school outcome than their component scores, had similar construct-level predictive validities to A-levels and Advanced Highers and are, therefore, probably not providing additional information over the simpler measures concerning construct-level predictive validity, although they may be better for those wishing to predict performance within medical school rather than for selection purposes.

### Predicting first year BMS examinations

In many ways the most important outcome in terms of medical student selection is performance in basic medical sciences examinations in the first year, as the end of the first year is mostly when failing medical students either have to leave the course or are required to repeat a year. Predicting first-year performance is, therefore, particularly important. The meta-regression contained three relevant construct-level predictive validities, and the meta-analytic estimate for A-levels of .809 (CI: .501 .935) is high, and is higher than for GCSEs/O-levels (.332; CI: .024 to .583) and for the sole aptitude test, UKCAT (.245; CI: .207 to .276).

### The Academic Backbone

Educational qualifications predict performance better in assessments earlier in training rather than later. That is hardly surprising, and to some extent reflects what we have elsewhere called the Academic Backbone [4], performance at each stage being built upon performance at previous stages. If educational qualifications predict, say, MRCP(UK) less well than they predict finals, that is in part because finals themselves are part of the prediction of performance at MRCP(UK). Likewise, GCSEs may not predict outcomes well, but they are good at predicting A-levels, which is perhaps their main role [54].

### How much can A-levels predict?

Using the meta-analytic first year BMS construct-level predictive validity estimate of .809, then 65% of the total, true variance in first year examination performance is accounted for by A-level performance, which clearly makes A-levels an important part of medical student selection. The estimate of .809 may itself be an under-estimate, in part because, as shown elsewhere [27], the measure we have called “EducationalAttainmentGCE” predicts outcome better than A-levels alone. That may be because A-levels are not always of equivalent difficulty [55], and better students may choose to take harder A-levels. The measure also includes General Studies which, contrary to popular belief, seems to be a separate and independent predictor of medical school performance [21]. Considering just A-levels, for which 65% of first year exam variance seems to be explained, the important corollary is that 35% of first year performance must be explained by something other than A-levels. Most of that 35% is unlikely to be assessed directly or indirectly by GCSEs or aptitude tests since both of those measures have little incremental validity over A-levels [21]. The most likely origin is in personality, motivation or other individual difference factors, although part of the explanation may also lie in the random, unpredictable events that occur in everyday life, including problems with peers, money, relationships, family or whatever, that are inherently unpredictable but can impact substantially on medical school performance, particularly in students who may recently have left home for the first time. Many such events cannot be predicted when selection takes place and, hence, any variance due to them cannot be taken into account by educational attainment or its correlates. Similar events which have happened before A-levels and selection could also be involved, lowering attained A-level grades, and when the impact of those events subsequently diminishes then students over-perform relative to what their A-levels might seem to have predicted. Whatever the nature of the missing variance, a major challenge has to be identifying the causes or the correlates of that additional variance, as it might account for a quarter or a third of the variance in first year medical school performance. In addition, because impacts on first year performance can subsequently be multiplied through the Academic Backbone with the accumulation of ‘medical capital’ [4], so small over- or under-achievements early in a career can potentially multiply as the medical course continues.

### The stability of construct-level predictive validity of educational achievement measures in the cohorts

The present studies took place in six cohorts of students who entered medical school from 1972 through to 2009. A remarkable finding is that all of the qualifications, be they A-levels, GCSEs/O-levels or aptitude tests, seem to predict at the same level across the entire temporal range of the cohorts. It might have been thought that changes in the nature of examinations such as A-levels, which have become less heavy on facts in recent years, might have altered their construct-level predictive validity. Medical school courses and assessments have also have become less fact heavy, with assessments now including OSCEs and other assessments of practical skills, communicative ability and so on, but despite that the predictive validity of the various qualifications seems to have remained equivalent.

### The role of GCSEs/O-levels

A recurrent theme in student selection is that GCSEs or O-levels may be better predictors of outcome than A-levels. As long ago as a GMC conference in 1973 it was reported that, “performance in the Second MB examination correlated better with GCE O level than with A level results” (p.7), with speculation that, “the O level correlation with future performance might be more accurate than the A level results, because at the latter stage the ‘heat was turned on’ for University entrance. [As a result] the A level results were based on factual knowledge and did not necessarily depend on greater intellectual capacity” [10] (pp. 7–8). The current meta-analysis provides no support for that argument in the undergraduate course, but it is striking that A-levels, like GCSEs/O-levels and aptitude tests, have similar construct-level predictive validities in both undergraduate and postgraduate assessments. Elsewhere we have noticed hints that GCSEs/O-levels may have additional predictive incremental value for predicting finals after taking A-levels and BMS performance into account [4], with the possibility that they are assessing something separate from the academic skills assessed in A-levels.

### Aptitude tests as predictors

The two tests of intellectual aptitude, UKCAT and AH5, predict undergraduate and postgraduate performance to similar extents with an overall construct-level predictive validity for undergraduate performance of .181, which is relatively low and is appreciably lower than for A-levels (.723) and GCSEs/O-levels (.359). In addition the incremental validities for AH5 [3] and UKCAT [21] are small once A-levels have been taken into account. UKCAT and similar tests may have some role to play in selection when there is strong range restriction on A-levels and other attainment tests, although the Sutton Trust reported that the SAT Reasoning test did not differentiate outcome in high-achieving university entrants with AAA grades [56] (pp.37-38). The UKCAT consortium is also currently piloting non-cognitive tests which may have additional predictive ability.

### What is the medical school applicant pool?

Our analyses have taken the pool of medical school applicants as being those who chose to apply, many of whom eventually attain quite low A-levels and other grades. Applying to medical school though is a choice, and there is no reason why candidates with substantially lower grades might not also choose to apply, particularly if medical schools were to suggest that there was a realistic chance that they might be admitted. The estimate of construct-level predictive validity for, say, A-levels is, therefore, an estimate given the applicants who actually applied. Were medical schools to suggest that applicants might be accepted with, say, the minimum matriculation grades of EE, then the variance in A-level grades of candidates would increase, resulting in the construct-level predictive validities being yet higher. Taking the concept to its extreme, were entrants of any intellectual ability to be allowed to enter, including those with minimal grades at GCSE (see the population distribution elsewhere [54]), then the construct-level predictive validity of educational attainment would probably rise close to one, as it also would were applicants to be admitted across the entire population range of intellectual ability.

### What happens to students who enter medical schools with substantially lower A-level grades?

One of the most interesting educational initiatives in UK medical education is the Extended Medical Degree Programme (EMDP) at King’s College, London [57-60], which admits students from low-achieving secondary schools who have A-level grades substantially below those normally required for medical school admission. Average grades initially were CCC (more recently rising to BBC), with BCC currently being the standard offer [61]. The study claimed that, “medical students can succeed without AAB at A level if these results were obtained from a low achieving [secondary] school” [57] (p.1113). The claim would be supported by the finding in the UKCAT-12 study that students attaining A-levels from under-achieving secondary schools subsequently do better at medical school [21], although the effect is relatively small (and the much larger HEFCE study found it to be of the order of one A-level grade, so that ABB from a lower achieving secondary school was equivalent to AAB from a higher achieving secondary school [62]). The effect of a low achieving secondary school is probably therefore too small to account for the claims made for the EMDP program, and potentially, therefore, is a challenge to the predictions made from construct-level predictive validity.

Formal statistical analyses have however suggested that EMDP students have a performance in finals which is about -.73 (CI: -.38 to −1.09) standard deviations below that of students on the five-year program [63]. In the present study, the meta-analytic estimate of construct-level predictive validity for finals in relation to A-levels is .625 (n = 5; CI: .449 to .754). Using a reliability of .905 for finals and .867 for A-levels (from the UKCAT-12 study), then the attenuated A-levels-Final correlation can be estimated at .553. A-levels in the UKCAT-12 applicants have a decensored mean of 29.01 (SD = 5.89), so that students with grades BBB, BBC, BCC and CCC are −.85, −1.19, −1.53 and −1.87 SDs below the mean without taking attenuation into account. Given the estimated A-levels-finals correlation of .553 they would be expected to score −.47, −.66, −.85 and −1.04 SDs below the mean in the finals assessment. The expected average for students with grades CCC to BBB is therefore about −.75, which is very close to the actual value of −.73. Were they admitted, entrants with grades of DDD or EEE would be expected to have mean scores −1.60 and −2.16 SDs below the mean.

In BMS examinations where conventional students show a retention rate of 97% (3% failing), EMDP students showed retention rates of 90% (10% failing) [57]. Retake rates for BMS exams are 15% in conventional students but 32% in EMDP students, with “A level chemistry and biology grades … of the EMDP students showing significant correlation with marks in the first year examinations” [57]. A variant on the calculation for finals can be used to predict these rates. Using a reliability for A-levels of .867, a reliability for a continuous overall BMS result of .904 (based on the UCLMS cohorts), and a meta-analytic construct-level predictive validity of .744 (n = 4; SD = .518 to .872), the attenuated predictor-outcome correlation is calculated as .659. A failure rate of 3% for conventional students implies that the cut-off is −1.88 SDs below the mean, and a retake rate of 15% implies a cutoff of −1.03 SDs. Failure rates for students with entry grades of BBB, BBC, BCC and CCC are then expected to be 9.3%, 13.6%, 19.1% and 25.8% the average of 17.0% being a little higher than the EMDP average of 10%. Likewise retake rates with grades of BBB, BBC, BCC and CCC are expected to be 31.7%, 40.0%, 48.8% and 57.7%, with the average of 44.6%, which again is a little higher than the EMDP’s rate of 32%. Were students to be admitted with grades of DDD or EEE then their failure rates would be expected to be 51% and 76%, with retake rates of 81% and 94%.

The calculation of construct-level predictive validity explicitly makes predictions outside of the normal range of the data for which the correlations were calculated. Although prediction outside of the range is often regarded as bad practice, it is precisely what construct-level predictive validity sets out to do, with a strong theoretical rationale and model behind it; and as the Statistical Appendix (Additional file 1) shows, the HSL method succeeds well at extrapolating correctly to the true figures in a simulation. The King’s EMDP data provide an independent validation of the predicted marks and failure rates. Failure rates and retake rates at BMS exams, and average marks at finals are predicted well from the estimates of construct-level predictive validity, being what would be expected given the A-level grades of the students. That provides confidence in the principle of calculating construct-level predictive validity as a basis for making selection decisions.

### A* grades at A-level

None of the studies described here had information on A* grades at A-level, which were first taken by students sitting A-levels in 2010. Few data have been published on A* grades in medical students, although in February 2013 data were published from Oxford, which is one of the most selective of UK medical schools. Of 2,054 applicants with A-levels, there were 16.7% with grades of less than AAA, 19.% with AAA, 22.4% with at least one A*, 16.9% with at least two A*s, and 24.8% with at least three A*s, with the proportions in those holding offers being 0.7%, 5.7%, 14.3%, 19.4% and 60.0% for grades AAA to A*A*A*. Scoring AAA = 30, AAA* = 32, AA*A* = 34 and A*A*A* = 36 [64], and using the estimates of reliability and construct-level predictive validity used for the King’s study (above), then compared with students scoring AAA, students with AAA*, AA*A* and A*A*A* grades are predicted to score .22, .45 and .67 SDs higher at BMS, and .19, .38 and .56 SDs higher at finals. Those predictions will soon be testable, in all medical schools and not just Oxford, and if correct then the utility of construct-level predictive validity will also be supported.

### Comparison with other studies of selection

This discussion is not the place for a full review of other studies which have assessed educational attainment measures and measures of intellectual aptitude as possible predictors of university and medical school performance. In US medical schools, there seems little doubt that MCAT [65] predicts medical school performance, with the Biological Sciences knowledge test having a higher prediction than the verbal reasoning (aptitude) test. For university admission in general, in the UK both ISPIUA [66,67] (in the 1960s) and the Sutton Trust SAT test [56,68] (in the 2000s) showed similar results, with A-levels being strong predictors of university performance and intellectual aptitude tests having little predictive value. The findings reported here are therefore compatible with other large-scale studies, albeit mostly not in medicine.

### Limitations of the present analysis

The present study is limited to a relatively small number of studies, albeit most include entrants to many UK medical schools, but longitudinal cohort studies are rare. The outcome variables are not always detailed, and postgraduate outcomes are restricted to the criteria of MRCP(UK) marks and Specialist Register entry. The statistical analyses also have to use estimates of some parameters such as reliabilities and selection ratios, and the unreliability of these may not have been taken fully into account. Future studies should examine a wider range of measures of clinical knowledge and performance. The outcomes considered here are almost entirely academic measures of success, and other, non-academic measures of clinical and professional performance in medical practice, would be desirable.

### What is the missing ‘dark variance’ of medical education?

Ultimately 100% of the true variance in medical school performance has to be accounted for, once unreliability, regression to the mean and right-censorship have been taken into account, even if some of that variance is sporadic (what one might call ‘deep chance’, to distinguish it from mere noise due to measurement error, and containing things such as the random, unpredictable events of every life, referred to earlier). The situation is akin to that currently being experienced in astrophysics, where the existence of ‘dark matter’ and ‘dark energy’ are inferred from the necessity, in what is effectively an accounting exercise, of accounting for the total mass of the universe and the expansion of the universe, all of which needs to be explained. Medical education also cannot account for all of the variation that needs accounting for, and selection of medical students can never be on a firm foundation without it being able to do so. Nevertheless, the present results provide robust support for the use of measures of educational attainment in student selection.

## Conclusions

Educational attainment at secondary school strongly predicts both undergraduate and postgraduate performance once attenuation due to unreliability, restriction of range and right censorship of educational qualifications has been taken into account. A-level grades in particular account for about 65% of true variance in first year performance, which strongly justifies the use of A-levels in student selection. If A-levels do account for 65% of variance, then the remaining 35% of variance must be accounted for by other, non-academic factors, measurement error, range restriction and right-censorship having already been taken into account). Just as in astrophysics, ‘dark matter’ and ‘dark energy’ are posited to balance various theoretical equations, so medical student selection must also have its ‘dark variance’, whose nature is not yet properly characterized, but explains perhaps a third of the variation in performance during training. Some variance probably relates to factors which are unpredictable at selection, such as illness or other life events, but some is probably also associated with factors such as personality, motivation or study skills.

## Endnote

^a^This may seem paradoxical at first glance. For the correction formulae of Hunter *et al*., when reliabilities are one and the selection ratio is one then the construct-level predictive validity is the same as the simple predictor-outcome correlation. Of necessity, construct-level predictive validity can only be higher or the same as simple predictor-outcome correlations (just as correlations disattenuated for lack of reliability must be higher than uncorrected correlations). Lower reliabilities and lower selection ratios therefore result in higher construct-level predictive validities. When reliabilities are low then there is less variance which is truly accounted for (but more that could be accounted for with a better test), and when selection ratios are low then the applicants have a much wider range of scores, both of which push up construct validity. The calculations for the standard Hunter, Schmidt and Le model are shown in Additional file 2, with a variety of situations with different values of the various parameters.

## Abbreviations

aAH5: Abbreviated AH5; AH5: Group test of General Intelligence [devised by Alice Heim]; A-level: Advanced level examinations; BMAT: BioMedical Admissions Test; BMS: Basic Medical Sciences; CI: Confidence interval; CLPV: Construct-level predictive validity; EM: Expectation-Minimization; EMDP: Extended Medical Degree Programme; GAMSAT: Graduate Medical School Admissions Test; GCE: General Certificate of Education; GCSE: General Certificate of Secondary Education; GMC: General Medical Council; GP: General practice/general practitioner; HSL: Hunter, Schmidt and Le model; ISPIUA: Investigation into supplementary predictive information for university admissions; LRMP: List of registered medical practitioners; MCAT: Medical College Admissions Test; MCMC: Markov chain Monte Carlo algorithm; MRCP(UK): Membership of the Royal College of Physicians of the United Kingdom; MSAT: Medical School Admissions Test; O-level: Ordinary level examinations; PACES: Practical assessment of clinical examination skills; POC: Predictor-outcome correlation; SD: Standard deviation; SPSS: Statistical Package for the Social Sciences; SQA: Scottish Qualifications Authority; UCL: University College London; UCLMS: University College London Medical School; UCMSM: University College and Middlesex School of Medicine; UK: United Kingdom; UKCAT: United Kingdom Clinical Aptitude Test; UMAT: Undergraduate Medicine and Health Sciences Admission Test; UMDS: United Medical and Dental Schools (Guy’s and St. Thomas’s).

## Competing interests

ICM’s university has received grants from the UKCAT Board during the conduct of the study, and he has on occasion provided advice to UKCAT. CD has received personal fees from UKCAT Board during the conduct of the study. SN is chair of the UKCAT Board, has sat on the UKCAT research working group during the time of this study, and has not received any personal financial reward or assistance with this study. SD reports that the University of Dundee is funded by UKCAT to manage and host one of the databases on which the part of this study was based, he has acted as a Board Member of the UKCAT consortium since 2008 and as lead of the UKCAT Research Panel since 2009. KW and HWWP declare that they have no competing interests.

## Authors’ contributions

The idea for the present study arose from discussions among ICM, CD, KW and HWWP, with the collaboration of SN and JSD. ICM, KW SN and JSD were curators of the various datasets assembled here. ICM and CD were commissioned by the UKCAT Consortium to analyze the UKCAT-12 data, and their institutions received a small amount of funding to support the work. Statistical analyses were mainly carried out by ICM with the assistance of CD, KW and HWWP. ICM wrote the first draft of the manuscript, which was reviewed by all authors, all of whom contributed to the final version. All authors read and approved the final manuscript.

## Supplementary Material

Additional file 1 **Statistical appendix: a) Using the MCMC method to extend the Hunter-Schmidt-Le method to include censoring and provide standard errors; and b) The estimation of reliabilities for various measures used in selection studies.** Click here for file

Additional file 2 This Excel spreadsheet carries out calculations for the standard method of Hunter, Schmidt and Le, and provides examples of effects when reliability and range restriction are varied with a fixed correlation between predictor and outcome in the restricted population. Click here for file
