# Supplementary material for: Construct-level predictive validity of educational attainment and intellectual aptitude tests in medical student selection: meta-regression of six UK longitudinal studies
Source: BMC Med. 2013 Nov 14;11:243. doi: 10.1186/1741-7015-11-243 (PMC3827328; doi:10.1186/1741-7015-11-243)

Statistical appendix

The Hunter-Schmidt-Le formulation of construct-level predictive validity in relation to unreliability and indirect range restriction.

Hunter, Schmidt and Le (HSL) [1] have formulated solutions to the problem of calculating construct-level predictive validity and we here will use the notation and the algorithms in their table 2. They differentiate between the actual selection scores, *X*, and their true underlying scores, *T,* which do not have measurement error, and the actual outcome measures, *Y*, and their true underlying performance scores, *P*, which do not have measurement error. They also distinguish between measures in the applicants, *a* (the candidates), and what they call the restricted population or the incumbents, *i* (the entrants or acceptances), for whom correlations and reliabilities are affected by range restriction. Their formulae require information about four separate parameters, all of which are in terms of actual measured scores (*X* and *Y*). Although HSL use both *r* and *ρ* (Greek lower-case rho) to symbolise correlations, for simplicity here we use *r* in all cases. The four parameters needed are:

- r_{XYi}, the correlation of the selection and outcome scores in the group of entrants.
- r_{YYi}, an estimate of the reliability of the outcome score, Y, in the group of entrants. Of necessity the reliability of the outcome score in the applicants cannot usually be known and has to be estimated from the reliability in the restricted group of entrants.
- u_X , a measure of the extent of range restriction in the selection measure, which can be calculated as SD_{Xi}/SD_{Xa}, the ratio of the standard deviations of the selection measure in the entrants divided by the standard deviation of the selection measure in the applicants.
- r_{XXa} , an estimate of the reliability of the selection measure in the applicants. Since the selection measure has been used in the unrestricted group of applicants, its reliability is calculated in that group.

In order to show the Hunter-Schmidt-Le method in action, and also to show its problems when data are right-censored, and a solution to the problem using the MCMC (Markov Chain Monte Carlo) method, we will create a large, simulated data set in which the true parameters are known, and hence the effectiveness of the methods for estimating the true parameters can be assessed.

The simulated data set.

Using Matlab, a set of 10,000 applicants was created. The scores for the Predictor had a normal distribution with a mean of 27 and an SD of 5, a score of 27 being halfway between ABB and AAB. The scores for the Outcome were normally distributed with a mean of 50 and SD of 10[a]. The reliability of the Predictor was set at 0.8, and since the reliability is the proportion of the predictor variance which is true variance, the SD of the true Predictor scores was set at sqrt(5 x .8) = 4.472. The reliability of the Outcome measure is slightly more complicated to calculate as a typical value *in the restricted population* is 0.9, but for the simulation an estimate is required in the applicants. The formula of step 2 in table 2 of HSL can be rearranged to estimate the reliability in the applicants, and for that an estimate is also needed of the selection ratio on the outcome variable, which later will be seen to be about .85, giving an estimate of the reliability in the applicants of .928. As a result the SD of the true Outcome scores was set at 9.633. The correlation of the true Predictor scores with the

---

[a] Note that the mean(SD) of 50(10) were used to distinguish them from the scores used in UKCAT which had a mean of 0 (SD 1) in *entrants.* The use of that scoring will be considered below when selection has been included in the simulation.

true Outcome scores (the Construct Validity) was set at 0.8. These values are shown in column (3) in table 1.

The random number generators in Matlab were used to generate the true scores of 10,000 notional applicants, and because of sampling error their actual values, shown in column (4), were not precisely those shown in column (3), although the correlation in particular is very similar (actual value to four decimal places = .7995). Results in table 1 for applicants are shown on a pink background and results for entrants, who will be considered later, are shown on a green background (corresponding in both cases to the colours in figures 4 and 5).

Column (5) in table 1 shows results for applicants but with error variance included, the unreliability of the predictor and outcome measure being shown lower in the table. Notice that the SD of the predictor is now 5, and likewise the SD of the outcomes is 10. The means are not altered by including the additional variance due to measurement error but, crucially, the correlation of the predictor with the outcome in the applicants is now substantially lower at .692 rather than the true correlation of .8.

The model thus far only considers correlations in applicants. However the reality of selection is that predictor-outcome selection is known only in entrants, and therefore selection itself has to be modelled. Although the major predictor of medical school selection is A-level grades, which is the particular predictor being considered here, it is usually the case, as HSL emphasise, that most selection in reality is indirect, involving a range of variables of which, in this case, A-levels is but one. To simulate that process a variable was created in applicants which correlated with the true score, but correlated only partly with A-levels[b]. For simplicity selection was modelled whereby the 50% or so of applicants were accepted who had scores above the mean of the indirect selection variable. The rows in green show equivalent statistics as for the 10,000 applicants but for the 5031 entrants.

Several effects are clear in the entrants. Even for the true scores in column (4) the predictor-outcome correlation is reduced from its correct value of .8 to a value of .708, showing the effect of restriction of range, the SD of true predictor scores being reduced from 4.48 to 3.36, and the SD of true outcome scores being reduced from 9.54 to 8.03. A key value in the Hunter-Schmidt-Le method is the *selection ratio*, the ratio of the SD of the predictor scores in entrants to that in applicants, and for the true scores it has a value of .750. The reliabilities of the true scores are all of course 1, in both applicants and entrants.

The HSL method requires four parameters and these are emphasised in table 1 in large, red, bold font, and with these values the construct-level predictive validity can be calculated using the formula provided by HSL. Construct-level predictive validity is shown in the bottom row of the table and for column (4) it is .801, and it therefore takes the range restriction in the entrants into account.

For real scores attenuated by error, as in column (5), the predictor-outcome correlation in entrants has fallen yet further compared with that for the true scores of .708, and is now .523. Once again that value, along with the selection ratio of .773, and the reliabilities of .700 and .893 can be entered into the HSL algorithm, and the construct-level predictive validity estimated. The estimated construct-level predictive validity of .793 is very close to that of the true value of 0.8, and hence the HSL method has successfully found the true construct-level predictive validity, despite range

[b] In practice it matters little what the correlation is. Exploration shows that if the correlation with A-levels is weak then the effect is to alter the selection ratio, and hence the effect gets taken into account. For simplicity we created a parallel score to the predictor, which since it had the same reliability as the predictor therefore correlated .8 with the actual predictor.

restriction and attenuation, and the reliability of the outcome measure and the predictor-outcome correlation being known only in entrants.

Medical school selection has the further problem of the right-censorship of predictor scores, as is clearly shown in figures 1 and 2 of the main paper. An important question therefore is whether the HSL method can reconstruct construct-level predictive validities in the presence of censorship. The simulation modelled censorship by setting all Predictor scores of greater than 30 as being exactly 30 (equivalent to AAA). Column (6) shows the consequences. Censorship has multiple effects on the results. Compared with attenuation alone, censorship reduces the predictor-outcome correlation further (in the applicants from .675 to .632) and particularly in the entrants (from .523 to .396). The SD of the predictor has also fallen, from 4.99 to 3.84 in applicants and from 3.86 to 2.08 in entrants, the combined effect being to reduce the selection ratio from .773 to .542. The reliability of the predictor has also fallen, in the applicants (from .799 to .750) and in the entrants (from .624 to .428). The consequence of all of these effects is that when the HSL algorithm is applied then the estimate of the construct-level predictive validity is 1.004, the exclamation mark in the table emphasising not only that the value is wrong (the true underlying construct-level predictive validity is 0.8) but also that the value is not possible, construct-level predictive validities, like correlations, having a maximum value of 1. It is clear that the HSL algorithm, while working well with normally distributed variables, produces erroneously high results when right-censorship is present.

A final problem for medical school selection is grouping of variables. Although many outcome variables measured in medical school are on a continuous scale, for many reasons such results are often only available as ordinal categories such as Honours, Pass, Resits and Fail, with disparate proportions in the groups. The same problem exists to some extent in predictor measures, and for instance although the calculations thus far have treated the predictor as if it is continuous, in practice A-level and other results are often only present in groups such as AAA, AAB, ABB, and so on, which are scored 30, 28, 26, etc.. To model the consequences of grouping of measures, the predictor was grouped into scores of 12, 14, 16, 18, 20, 22, 24, 26, 28 and 30, equivalent to DDD (or less) to AAA. The 5031 outcome scores for entrants were coded arbitrarily into four groups, with 63 (1.3%) in the lowest category (equivalent to fail), 13.8% in the resit category, 80.6% in the pass category, and 215 (4.3%) in the Honours category, all of the proportions being broadly comparable to those actually found in medical school. The four groups were scored 1, 2, 3 and 4 for statistical analysis, with the ten Predictor groups scored from 12 to 30. The data are summarised in table 2, which at the top shows, for *entrants*, the outcomes in relation to the predictor, with the correlation being clearly visible. At the bottom is shown a similar table for *applicants,* with the outcomes in inverted commas to indicate that they are the results which would have been achieved, given the model, had those applicants been accepted.

The data of table 2 could be treated as continuous and conventional correlations calculated, and those correlations are shown in table 1. The addition of grouping has reduced the predictor-outcome correlations yet further in both applicants, particularly in entrants, where it is now down to .312. The grouping in particular has also reduced the reliability of both the predictor and outcome measures. Attempting to use the HSL algorithm results in multiple errors, with a very low negative value of -0.988 for rXXa at step 2, which then produces a calculation error at step 3 since the square-root of the negative reliability is needed. No construct-level predictive validity can therefore be calculated for these values.

MCMC estimation of construct-level predictive validity.

Although the HSL algorithm works well for data which are normally distributed it clearly fails when there is censorship of the data, as occurs with medical school selection data. The means, standard deviations and correlation for bivariate censored data can be estimated straightforwardly using the MCMC (Monte Carlo Markov Chain) [2]. The programs used the DRAM adaptation of MCMC[3], available for Matlab from Dr Marko Laine of the University of Helsinki (see helios.fmi.fi/~lainema/mcmc/ , helios.fmi.fi/~lainema/mcmc/mcmcstat.zip and helios.fmi.fi/~lainema/dram/). A worked example for right-censored and grouped data can be found in the Additional Data for our Academic Backbone paper [4]. The present case is similar but with a little additional complexity. The primary data for estimating construct-level predictive validity is the bivariate distribution in the entrants of predictor in relation to outcome (see the yellow area in the upper part of table 2), for which five parameters need to be estimated (two means, two standard deviations and the correlation). In addition data on applicants is required (see the yellow boxes in the lower part of table 2, which is also censored and estimates are required of the mean and standard deviation.

The Outcome data in table 2 have been divided into four groups, and although these groups are ordinal there is no reason to believe that the scale values attached to them of 1, 2, 3 and 4 correspond to true distances between them. The MCMC program therefore treats them as ordinal, calculating the area under a standard normal curve which would be associated with their relative proportions. In effect that is the same as the methods of tetrachoric, biserial and polychoric correlations (and in the present case could be called a polyserial correlation, the Predictor being treated as on an interval scale whereas there are several ordinal groups in the Outcome).

The MCMC algorithm calculates an estimate of the seven parameters at each step in the 'chain', the chain typically being 5,000 or 10,000 steps long, by which time ergodic stability is usually reached, and is checked by examining a plot of the parameters against step number, as in figure 1, which is based on 10,000 steps. It can be seen that the program 'hunts around' for a while, and then reaches stability after about 1000 or so steps. Estimates of the parameters are calculated on the basis of the mean of the estimates in the last 2000 steps in the chain (and it can be seen in this case that equivalent estimates would have been obtained if the last 2000 of only 5000 steps had been used). Importantly, an estimate of the standard error and 95% confidence interval of the estimates can be obtained by calculating the standard deviation of the last 2000 steps (for the standard error), or by looking at the 2.5th and 97.5th percentiles for the confidence intervals (which need not be symmetric around the estimate).

Column (8) of table 1 shows the estimates of the various parameters calculated using the MCMC algorithm. Confidence intervals are not shown, but are generally small and occasional examples will be given here. The mean of the predictor in the applicants is estimated at 27.07 (CI 26.97 to 27.19), which is higher than the raw value in column (7), and importantly is the same as the true estimate in column (5). Likewise the mean predictor in entrants, for which the raw value in column (7) is 28.59, is estimated by MCMC as 30.04 (CI 29.88 to 30.19), which is similar to the true value of 30.21 in column (5). Similarly the MCMC estimates of the standard deviations in applicants and entrants of 5.01 and 3.64 are substantially larger than the raw estimates of 3.88 and 2.15 in column (7), but close to the true actual values of 5 and 3.86 in column (5). The predictor-outcome correlation of .463 has a confidence interval of .428 to .493, and hence is slightly lower than the actual value of .523 shown in column (5).

The HSL algorithm requires four parameters, one of which, the predictor-outcome correlation of .463 is estimated directly by the MCMC algorithm. A second parameter, the selection ratio, is the ratio of the estimates of the standard deviation of the predictor in the applicants and the entrants. It

can be calculated from the two estimates of the standard deviation as 3.64/5.01 = .727. It also is slightly lower than the actual selection ratio of .773 in column (5). Of particular importance is that the selection ratio *can be calculated for every step in the chain*. Considering the last 2000 steps, the average value of the selection ratio is .727, and its 95% CI is .702 to .751. Confidence intervals can therefore be set on parameters derived from other parameters estimated in the chain.

The final two values required by the HSL algorithm are the reliabilities of the predictor and the outcome. Since both the predictor has been corrected for right-censorship and the outcome for reductions in correlation due to the data being ordinal, the appropriate estimates of the reliability are the true values of 0.8 and 0.9. needs also to be corrected in the same way. With real, empirical data these need to be calculated independently, and if necessary should take into account grouping, right-censorship and the ordinal nature of variables, and that will be described in more detail below.

Given the selection ratio, the predictor-outcome correlation, and the two estimates of reliability, the HSL algorithm can be calculated *for every step in the chain*. Figure 2 shows construct-level predictive validity plotted against selection ratio for the 10,000 steps of the chain. The overall estimate of the construct-level predictive validity is .776, with a 95% CI of .731 to .821, which includes the known, true value of 0.8. Figure 2 shows that there is a slight negative correlation, as would be expected, between estimates of selection ratio and construct-level predictive validity. The MCMC algorithm, correcting estimates of standard deviations and correlations for right-censorship, grouping or being ordinal, and then followed by the application of the HSL algorithm has successfully retrieved the true construct-level predictive validity from the much restricted data set shown in table 2. That gives faith in the statistical method as a whole.

Standing back from the details of the calculations, a striking feature of table 1 is how an apparently robust construct-level predictive validity of 0.8, with the Predictor accounting for 64% of the variance in Outcome is progressively reduced. For the attenuated, censored, ordinal types of data which are typical of the information available in medical student selection studies, the Outcome is correlating only .312 with the Predictor, apparently accounting for less than 10% of the variance in the Outcome. The value of .312 would be an accurate description were all that was required was an empirical prediction in entrants of how they would perform in medical school assessments. Such a correlation is however not a useful value for selectors who wish instead to select students who will respond well in medical school. Table 2 shows, in the bottom row, the percentage of students at various levels of the Predictor who for these parameters would be expected to fail or do resits at medical school, were they to have been admitted. The challenge for selection studies is therefore to estimate the true Predictor-Outcome correlation in applicants, the construct-level predictive validity, for that is the theoretical justification for using the Predictor as a selection tool, even when the Predictor-Outcome correlation in entrants might seem to be low.

The reliability of outcome and predictor measures.

The HSL method for calculating construct-level predictive validity requires estimates of the reliability of the various outcome measures, and these are often not readily available. In meta-analysis in situations where reliabilities are not available it is conventional to use averaged estimates from similar tests, and that strategy has been carried out here. Table 3 summarises the reliability estimates used in the various calculations of construct-level predictive validity for the different measures in each cohort.

Considering the individual outcome measures separately:

The reliability of basic medical science examinations. The only study for which estimates of the reliability of BMS examinations could be made was the UCLMS Cohort Study. For a composite of first and second year BMS examinations the reliability was .904. Using the Spearman-Brown formula it can be estimated that the reliability of the sum of the two first year examinations was .825. No other reliabilities for BMS assessments are available and therefore we have used .904 for overall BMS results, and .825 for the reliability of BMS first or second year results, in whichever studies they occurred.

The reliability of clinical examinations and medical school finals. The most detailed results were available for the 1985 and 1980 cohorts where information was available on University of London finals, with the grand total of marks, which were standardised and aggregated across 25 separate measures in five different clinical areas[5], being normally distributedE. For the 1985 cohort, Cronbach's alpha was .897, and for the 1980 cohort Cronbach's alpha was .913. In the UCLMS Cohorts [4] the reliability of summed clinical assessments across the three years was .913, giving an estimate of .778 for the final year alone, using the Spearman-Brown formula. No reliability was available for the 1990 Cohort or the Westminster Cohort and the average of the 1985 and 1980 cohorts, .905, was used in each case.

The Reliability of MRCP(UK) examinations. The UCLMS cohort took MRCP(UK) Part 1 from about 2007 onwards. The reliability of the Part 1 examination overall is of the order of .912 [6], but that figure is based on all candidates, UK candidates having a higher mean score and a somewhat lower SD, meaning that the reliability will be rather lower in them. For a randomly chosen diet of the current Part 1 examination the reliability was .918 overall and .894 for UK graduates only, the UK graduates having a raw score SD of 19.2 compared with 22.2 for all candidates. The reliability estimate of .89 will therefore be used here for Part 1 examinations in the UCLMS cohort. The 1990 cohort took the old style Part 1 examinations, for which a mean reliability was .865[7]. Information was not available on UK and non-UK graduates, but assuming that the SDs were lower in the UK graduates by a similar proportion to that for the new form of the exam (i.e. 19.2 to 22.2), then the reliability of UK graduates would be about .82, and that value will be used here.

The reliability of the new Part 2 examination overall is of the order of .828 [6]. In two randomly chosen diets of Part 2 there was no evidence of differences in SD between UK and non-UK candidates, and therefore the estimate of .828 is used for Part 2 reliability in the UCLMS cohort. The reliability of the old Part 2 written examination, taken by the 1990 cohort, has not been published, but a review of the diets from 1979 to 2000[8] suggests it was of the order of .66, the lowish value primarily reflecting a relatively small number of items, which was one of the reasons for the introduction of the longer, new format, exam. The value of .66 will be used here for the 1990 Cohort.

The reliability of the MRCP(UK) PACES exam has been estimated elsewhere at .82[9], although for technical reasons that may be a little high. The estimate of .82 will nevertheless be used here for the UCLMS cohort. There are no estimates of the reliability of the old Part 2 Clinical examination, which was taken by the 1990 Cohort, but given the reduced reliability of the old Part 2 Written examination (.66 compared with a current .828), and the then structure of the Clinical examination, it seems unlikely that the reliability could be higher than 0.7. That value will therefore be used for the 1990 Cohort Study.

Reliability of being on the Specialist Register. This is a binary measure, and there seems little way of estimating the reliability beyond a guesstimate. A value of .7 will be used, although that is clearly arbitrary. If there are concerns about it then sensitivity analyses can be carried out.

Reliability of three best A-levels. The most important predictor variable is three best A-levels. Although there is some discussion in the literature of the problems of estimating the reliability of A-levels [10], and despite the fact that using three best A-levels is a standard method both in research and in medical student selection, we know of no formal estimates of reliability. Because the HSL method requires the reliability in applicants, the reliability could only be calculated in proper selection studies with detailed information on applicants. In view of the potential interest in estimates of the reliability of educational attainment measures in a recent cohort of medical school applicants, the detailed estimates for the UKCAT-12 cohort are shown in table 4. The table also shows how decensoring the correlations results in a higher alpha estimate than does the standard method using raw scores.

Detailed A-level results on applicants were available for the UKCAT-12, 1990 and 1980 Cohort Studies. A Matlab program was written which took the three best A-level grades for each participant, and then randomised these as A-level 1, A-level 2 and A-level 3. Since the three A-levels were randomised the pairs of results were then stacked to give a single pair of grades. A conventional Cronbach's alpha could then have been calculated, except that in many cases there were ceiling effects on A-level grades, and therefore correlations between the pairs of A-levels were calculated after correcting for right-censorship, using an MCMC method. Given the correlation between a (random) pair of A-levels then the reliability of the total based on three best A-levels could be estimated using the Spearman-Brown formula.

The reliability of three A-levels in applicants for the 1981 and 1991 cohorts were .707 and .686, and for UKCAT-12 were .867. In passing we also note that we had data on two other very large datasets, of all UCAS applicants for *any* subject in 2003-5 [11] which gave a corrected reliability of .835, and the Youth Cohort Study [12], which gave an estimate of .829. The UCAS medical school applicants had a reliability of .871, somewhat higher than for applicants in general. Taking the results overall it would seem that reliability has increased somewhat over the years, and looking in more detail at the data suggested that might reflect less variable performance within candidates, large differences such as AEE or AAD becoming less common.

Estimates for the 1985 and Westminster cohorts were based on the mean of the 1980 and 1990 cohorts, which was .697. The UCLMS cohort had an entry date slightly earlier than that of the UCAS 2003-5 medical school applicants, and therefore an estimate of .845 was used, interpolating between the 1990 Cohort and the UCAS 2003-5 reliabilities.

Reliability of AS-levels. Detailed AS-level results were available for UKCAT-12. Reliability was calculated as for A-levels, except that the four best A-levels were used, all possible random pairs were looked at, corrected for right-censorship, and the Spearman-Brown formula applied. The estimate of reliability in applicants was .873, very similar to that for A-levels.

Reliability of GCSEs and O-levels. A typical number of GCSEs taken in UKCAT-12 was nine, and therefore reliability was estimated for the nine highest GCSEs, using the method described above, correcting for right-censorship to all possible random pairs and then applying the Spearman=Brown formula, giving an estimate of .906. No other reliability estimates of GCSEs or O-levels were available and therefore the value of .906 was used for all other studies as well. It should be noted that the introduction of the A* grade should not affect the reliability estimate as the correction for right censorship in effect takes that into account.

Reliability of Highers, 'Highers Plus' and Advanced Highers. The reliability of best five Highers and best five at 'Highers Plus' was calculated in the same way as for best A-levels, with reliabilities of

.834 and .832. Estimating the reliability of the best Advanced Higher was not as straightforward, since there is only a single result. However sufficient candidates take two Advanced Highers that it is possible to calculate the reliability of the best two Advanced Highers, and then use the Spearman-Brown formula to calculate the reliability of a single best Advanced Highers, which was .730.

Reliability of Educational Attainment Measures. Calculating the reliability of the Educational Attainment measures was not straightforward. The GCE measure is a composite primarily of the best A-levels, AS-levels and GCSEs, which have an average reliability of .882. The three measures correlate somewhat, and therefore the reliability was calculated on the basis of two independent measures, which using the Spearman-Brown formula gives a reliability of .937. A similar method for the SCE qualifications gives an estimate of the reliability of .913.

Reliability of UKCAT total score. The UKCAT Annual Reports provided estimates of the reliability of the total score in medical school applicants of .87 and .86 [13;14], and so a mean value of .865 was used.

Reliability of AH5 and aAH5 tests.

Item scores were no longer available for the AH5 and aAH5 tests used in the Cohort Studies, so that Cronbach's alpha could not be calculated directly. However the scores obtained in the Westminster Study could be directly compared to those in the test manual [15]. The total score had a mean of 40.4 (SD=7.73, n=505) which was similar to the values for other groups of students in the manual ("University Students", mean=39.06, SD=8.26, n=946; "medical students", mean=37.49, SD=7.53, n=866; "Post-graduate students in Education: Science", mean=39.01, SD=7.97, n=363). The Verbal and Spatial sub-scores also had a correlation of .488, which is similar to the correlations of .49, .52, .50, .62 and .52 reported in the manual. Overall the scores of the Westminster students are similar to those Heim reported, and therefore it is reasonable to use the three estimates in the manual of test-retest reliability of .84, .82 and .89, giving a mean of .85, a value which is similar to many other IQ tests, particularly those which do not have participants across the entire population range of ability.

In the applicants in the 1990 Cohort Study, the mean score on the aAH5, a test of half the length of the full AH5, was 20.08 (SD=4.41, n=929). Equivalent scores for a full-length test would be 40.16 (SD=8.82), which are equivalent to those reported above. The correlation for the verbal and spatial subtests was .252, which is a little lower than the expected correlation of .37, although it is subject to some uncertainty. If as above the reliability of the total score on the AH5 is taken as 0.85, the using the Spearman-Brown formula, the expected reliability for the total score on the aAH5, a test of half the length, is 0.74.

Typical reliabilities of predictors and outcomes. Table 3 shows ten independent empirical estimates of the reliability of predictors, which have a mean value of .815. There are seven independent empirically-based estimates of the reliability of outcomes (including the five external estimates of the reliability of the old and current MRCP(UK) Parts 1 and 2, and the current MRCP(UK) clinical exam), which together have a mean reliability of .834.

Corrections of reliability coefficient when using ordinal measures. The reliabilities reported above are based on continuous, approximately normally distributed measures (such as year totals in undergraduate performance), and they have been corrected for right-censorship due to ceiling effects.

In some of the present cohorts the outcome measures are not continuous, normally distributed scores but are discrete, ordinal categories, a situation which is often the case in education research, with typical categories being Fail, Resit, Pass and Honours (or even sometimes, Fail and Pass). If such categories are used, and scored as say 1,2,3,4 or 0,1 then as is typical in contingency tables, the reliability is less than would be expected due to the categorisation. It is possible to estimate the reliability of such scores numerically using the expected proportions under a bivariate normal distribution with the known reliability. For instance, for the 1990 Cohort Study the overall BMS score was on a four-point scales, the categories proportions of .055, .043, .216 and .687. With a true (continuous) reliability of .904, the expected reliability for the four-point scale with those proportions is .83. Similarly for the simulated data above, in table 2, where the true reliability of the outcome measure in entrants is 0.9, the four categories are in proportions .013, .138, .806 and .043, the estimate reliability is .770, which is similar to the empirical estimate of the reliability of the outcome in column 7 of .693.

Having described how reliabilities can be corrected for using only small numbers of categories (and it can be useful in interpreting studies where only simple Pearson correlations are provided with such scales), it also needs emphasising that in the present analysis such corrections are *not* needed. Where scales have only small numbers of ordinal categories then the analyses of construct-level predictive validity, as in the simulated example above, take the ordinal nature of the data into account, and in effect provide the equivalent of tetrachoric, biserial or polychoric correlations. The appropriate reliabilities for those estimates are therefore the ones based on continuous, normally distributed variables, since the latent scale on which the construct-level predictive validities is estimated is continuous and normally distributed.

Reference List

(1) Emery JL, Bell JF, Emer, Rodeiro CLV. The BioMedical Admissions Test for medical student selection: Issues of fairness and bias. *Medical Teacher* 2011; 33:62-71.

(2) Press WH, Teukolsky SA, Vetterling WT, Flannery BP. Numerical Recipes: The art of scientific computing (Third edition). New York: Cambridge University Press; 2013.

(3) Haario H, Laine M, Mira A, Saksman E. DRAM: Efficient adaptive MCMC. *Statistics and Computing* 2006; 16(4):339-354.

(4) McManus IC, Woolf K, Dacre J, Paice E. The academic backbone: Longitudinal continuities in educational achievement from secondary school and medical school to MRCP(UK) and the Specialist Register in UK medical students and doctors. *Submitted* 2013.

(5) McManus IC, Richards P, Winder BC, Sproston KA. Final examination performance of students from ethnic minorities. *Medical Education* 1996; 30:195-200.

(6) Tighe J, McManus IC, Dewhurst NG, Chis L, Mucklow J. The Standard Error of Measurement is a more appropriate measure of quality in postgraduate medical assessments than is reliability: An analysis of MRCP(UK) written examinations. *BMC Medical Education (www biomedccentral com/1472-6920/10/40)* 2010; 10:40.

(7) McManus IC, Mooney-Somers J, Dacre JE, Vale JA. Reliability of the MRCP(UK) Part I Examination, 1984-2001. *Medical Education* 2003; 37:609-611.

(8) McManus IC. Reliability of the MRCP Part 2 examiation, 1979-2000. London: Unpublished manuscript; 2002.

(9) McManus IC, Thompson M, Mollon J. Assessment of examiner leniency and stringency ('hawk-dove effect') in the MRCP(UK) clinical examination (PACES) using multi-facet Rasch modelling. *BMC Medical Education* 2006; 6: 42 (http://www.biomedcentral.com/1472-6920/6/42/abstract).

(10) Bramley T, Dhawan V. Estimates of reliability of qualifications. Coventry: Office of Qualifications and Examinations Regulation (http://www.ofqual.gov.uk/files/reliability/11-03-16-Estimates-of-Reliability-of-qualifications.pdf); 2011.

(11) McManus IC, Woolf K, Dacre J. The educational background and qualifications of UK medical students from ethnic minorities. *BMC Medical Education* 2008; 8: 21 (http://www.biomedcentral.com/1472-6920/8/21).

(12) Russell N, Phelps A. Youth Cohort Study: Cohort 10 Sweep 1 (C10S1). Technical Report. http://www.data-archive.ac.uk/doc/4571%5Cmrdoc%5Cpdf%5C4571userguide.pdf: 2001.

(13) UKCAT. UKCAT: 2007 Annual Report. Cambridge: United Kngdom Clinical Aptitude Test (available at http://www.ukcat.ac.uk/pdf/UKCAT Annual Report 2007.pdf); 2008.

(14) UKCAT. UKCAT 2008 Annual Report. Nottingham: UKCAT; 2008.

(15) Heim AW. AH5 group test of high-grade intelligence. Windsor: NFER-Nelson; 1968.

Table 1: A summary of the simulated data in the statistical appendix which is used to demonstrate that as long as the data are normally distributed the HSE algorithm successfully retrieves the true construct validity (columns 3 and 4) despite attenuation and range restriction (see column 5). However when there is strong right-censorship of the predictor the HSE algorithm either over-estimates the construct validity or it fails entirely due to numerical problems (columns 6 and 7). Grouping of the data also adds additional problems. The final column (8) shows the estimates of the various parameters, including the construct validity, when MCMC estimation is used to calculate the underlying variances and correlations of a distribution which is both censored and grouped. See text for further details. For the applicants a total of 10,000 individuals were generated, of whom 5031 were entrants.

(1)	Group (2)	Parameters (3)	(4)	**(5)**	(6)	(7)	(8)
True Scores			True scores	True scores	True scores	True scores	True scores
Attenuation of Predictor				**Attenuated**	Attenuated	Attenuated	Attenuated
Attenuation of Outcome				**Attenuated**	Attenuated	Attenuated	Attenuated
Censorship of Predictor					Censored	Censored	Censored
Predictor						Grouped	Grouped
Outcome						Grouped	Ordinal
Estimation			Pearson corr.	Pearson corr.	Pearson corr.	Pearson corr.	MCMC
Predictor-Outcome Correlation	Applicants	.8	.800	**.683**	.649	.601	**
	Entrants	-	***.708***	***.523***	***.396***	***.312***	***.463***
SD Predictor	Applicants	4.47	4.48	**4.99**	3.84	3.88	5.01
	Entrants	-	*3.36*	***3.86***	*2.08*	*2.15*	*3.64*
	Selection Ratio	-	***.750***	***.773***	***.542***	***.554***	***.727***
SD Outcome	Applicants	9.63	9.54	**9.94**	9.94	.717[+]	**
	Entrants	-	*8.03*	***8.31***	*8.31*	*.466*[+]	*1.001*[++]
Mean Predictor	Applicants	27	27.04	**27.05**	26.20	26.20	27.07
	Entrants	-	*30.01*	***30.21***	*28.58*	*28.59*	*30.04*
Mean Outcome	Applicants	50	50.08	**50.09**	50.07	2.53[+]	**
	Entrants	-	*55.05*	***55.52***	*55.52*	*2.88*[+]	*-.012*[++]
Reliability of Predictor*	Applicants	.8	**[1]**	**.799**	**.750**	**.760**	**[.8]**
	Entrants	-	*[1]*	***.624***	*.428*	*.390*	**
Reliability of Outcome*	Applicants	-	[1]	**.926**	.926	.819	**
	Entrants	*.9*	***[1]***	***.893***	***.893***	***.693***	***[.9]***
Construct validity			**.801**	**.793**	**1.004**[#]	**#**	**.776**

*estimated empirically for the simulated data by creating a parallel test with the same true values

[#] The value is out of the possible range for a construct validity or is incalculable (e.g. requires square-root of negative number)

[+] Values are on different scales to those in columns 3 to 6 (scored 1 to 4 for Fail to Honours) and therefore cannot be directly compared.

[++] Values are on different scales to those in columns 3 to 7, and are standardised to mean of zero and standard deviation of 1, and therefore cannot be directly compared.

** Not estimated by the MCMC algorithm

Table 2: The relationship between the predictor variable and the outcome variable for the simulated data provided in the appendix in which the true construct validity is 0.8, the reliability of the predictor in applicants is 0.8 and the reliability of the outcome measure is 0.9 in entrants. The Predictor has been divided into 10 categories, corresponding to A-level grades, and there is strong right-censorship, many applicants having AAA grades who would have had higher grades had they been available. The outcome measure is continuous in the simulation but has been reduced to four categories, labelled as Fail, Resit, Pass and Honours, in arbitrarily chosen proportions which are compatible with the values found in medical schools. The top half of the table shows the Predictor-Outcome correlation in medical school entrants. The lower line of the top half also shows the proportions of applicants who would be expected to be accepted. The lower half of the table shows the Predictor-Outcome relationship which would be expected were all of the applicants to enter medical school. The overall distribution of Predictors is shown in the penultimate line, and is typical of medical school applications, with strong right-censorship but a long left tail. The bottom line shows the proportion of individuals at each Predictor grade who would be expected to fail or to resit given the construct validity. The cells shown in bold with a yellow background are the numbers which are input to the MCMC algorithm, the upper cells being used to calculate the means, standard deviations and correlation in entrants, and the lower cells being used to calculate the mean and standard deviation in applicants. See the text for further details.

		**Predictor**										
	**Outcome**	DDD	DDC	DCC	CCC	BCC	BBC	BBB	ABB	AAB	AAA	Total
**Entrants**	Fail	**0**	**0**	**0**	**0**	**1**	**12**	**13**	**13**	**16**	**8**	*63*
	Resit	**0**	**0**	**0**	**1**	**10**	**43**	**85**	**159**	**155**	**243**	*696*
	Pass	**0**	**0**	**0**	**1**	**10**	**53**	**195**	**428**	**738**	**2,632**	*4,057*
	Honours	**0**	**0**	**0**	**0**	**0**	**0**	**1**	**2**	**4**	**208**	*215*
	Total	*0*	*0*	*0*	*2*	*21*	*108*	*294*	*602*	*913*	*3091*	*5,031*
	% accepted	0%	0%	0%	0.6%	3.6%	10.9%	22.0%	39.4%	60.5%	88.0%	*50.3%*
**Applicants**	‘Fail’	22	56	77	160	209	229	184	97	53	14	*1,101*
	‘Resit’	2	7	31	135	256	470	570	564	342	334	*2,711*
	‘Pass’	0	0	5	34	119	294	584	868	1,113	2,951	*5,968*
	‘Honours’	0	0	0	0	0	0	1	2	5	212	*220*
	Total	***24***	***63***	***113***	***329***	***584***	***993***	***1,339***	***1,531***	***1,513***	***3,511***	*10,000*
	% Fail-resit	*100%*	*100%*	*96%*	*90%*	*80%*	*70%*	*56%*	*43%*	*26%*	*10%*	*38%*

Table 3: Estimates of reliabilities of predictors and outcomes used in the calculations of construct validity. Estimates in square brackets are based on estimates in other studies which are broadly equivalent, and further details are given in the text.

	UKCAT-12	UCLMS	1990	1985	1980	Westminster
Three best A-levels	**.867**	**.845**	**.686**	**[.697]**	**.707**	**[.697]**
Four best AS-levels	**.873**	n/a	n/a	n/a	n/a	n/a
Nine best GCSEs/O-levels	**.906**	**[.906]**	**[.906]**	**[.906]**	**[.906]**	n/a
Five best Highers	**.834**	n/a	n/a	n/a	n/a	n/a
Five best 'Highers Plus'	**.832**	n/a	n/a	n/a	n/a	n/a
Five best Advanced Highers	**.730**	n/a	n/a	n/a	n/a	n/a
Educational attainment GCE	**[.937]**	n/a	n/a	n/a	n/a	n/a
Educational attainment SQA	[.913]	n/a	n/a	n/a	n/a	n/a
UKCAT total score	**.865**	n/a	n/a	n/a	n/a	n/a
AH5	n/a	n/a	**[.74]**	n/a	n/a	**.85**
BMS overall	n/a	**.904**	**[.904]**	**[.904]**	**[.904]**	n/a
BMS1	**[.825]**	**[.825^{+}]**	n/a	n/a	**[.825]**	n/a
BMS2	n/a	**[.825^{+}]**	n/a	n/a	**[.825]**	n/a
Clinical exams overall	n/a	**.913**	n/a	n/a	n/a	n/a
Final examination	n/a	**[.778^{+}]**	**[.905]**	**.897**	**.913**	**[.905]**
MRCP(UK) Part 1	n/a	**[.890**]**	**[.820**]**	n/a	n/a	n/a
MRCP(UK) Part 2	n/a	**[.828**]**	**[.660**]**	n/a	n/a	n/a
MRCP(UK) Clinical	n/a	**[.820**]**	**[.7*]**	n/a	n/a	n/a
Specialist Register	n/a	**[.7*]**	**[.7*]**	**[.7*]**	**[.7*]**	**[.7*]**

$^{+}$ Estimated using the Spearman-Brown formula from another estimate in the same study. See text for details.

* Guesstimate – see text for details.

** calculated externally – see text for details.

Table 4: Reliabilities of Educational Attainments in UKCAT applicants. See text for description of MCMC method used to estimate reliability of the various measures.

Measure	Standard alpha	Decensored alpha	Mean (Decens)	SD (Decens)	N exams (candidates)	Decens r for a pair
3 best A-levels	.8080	**.867** (.8634; .8697)	26.55	4.26	68232 (22744)	.6899
4 best AS-levels	.7653	**.8279** (.8248; .8308)	34.02	5.93	95766 (20019)	.5460
9 best GCSEs	.8832	**.9063** (.9054; .9070)	44.64	6.02	588600 (16350)	.5180
5 best Highers	.7538	**.8396** (.8318; .8471)	46.54	4.49	25820 (2582)	.5116
5 best HighersPlus	.7992	**.8322** (.8265; .8378)	44.04	4.73	25390 (2539)	.4981
2 best Advanced Highers	.6626	**.7303** (.701; .7579)	16.16	3.069	2012 (2012)	.5754

Figure 1: The MCMC chain for 10,000 steps, for estimating the mean and standard deviation of the Predictor in entrants (X), the mean and standard deviation of the Outcome in entrants (Y), and their correlation (r), as well as the mean and standard deviation of the Predictor in applicants (X unrestricted).

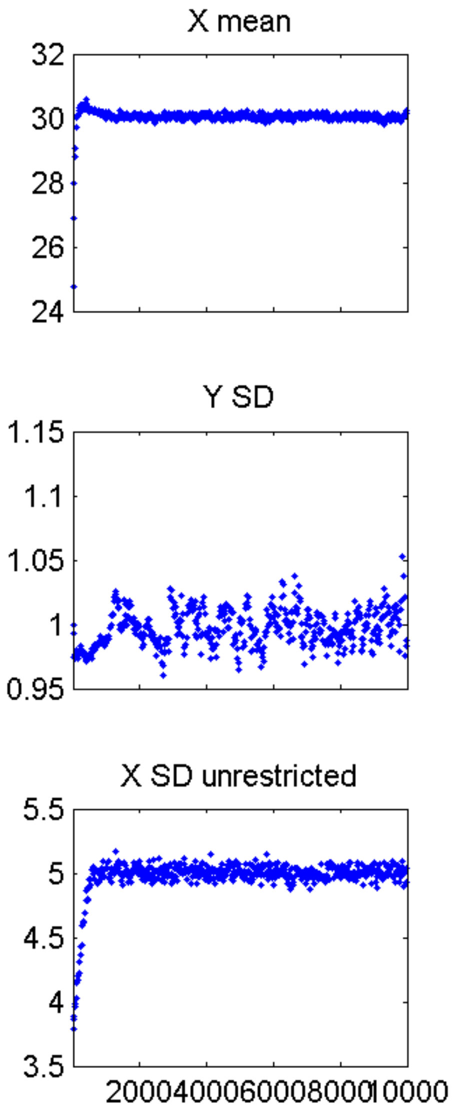

Figure 2: Estimates for the 10,000 steps of the MCMC chain of u(x), the selection ratio of the Predictor variable in Entrants to Applicants (x axis), and of the estimated construct validity (y axis). The red arrow shows the starting point of the chain (coloured dark blue), and the other colours indicate the passage of the chain in 'jet stream' colours, hotter colours being at the end.

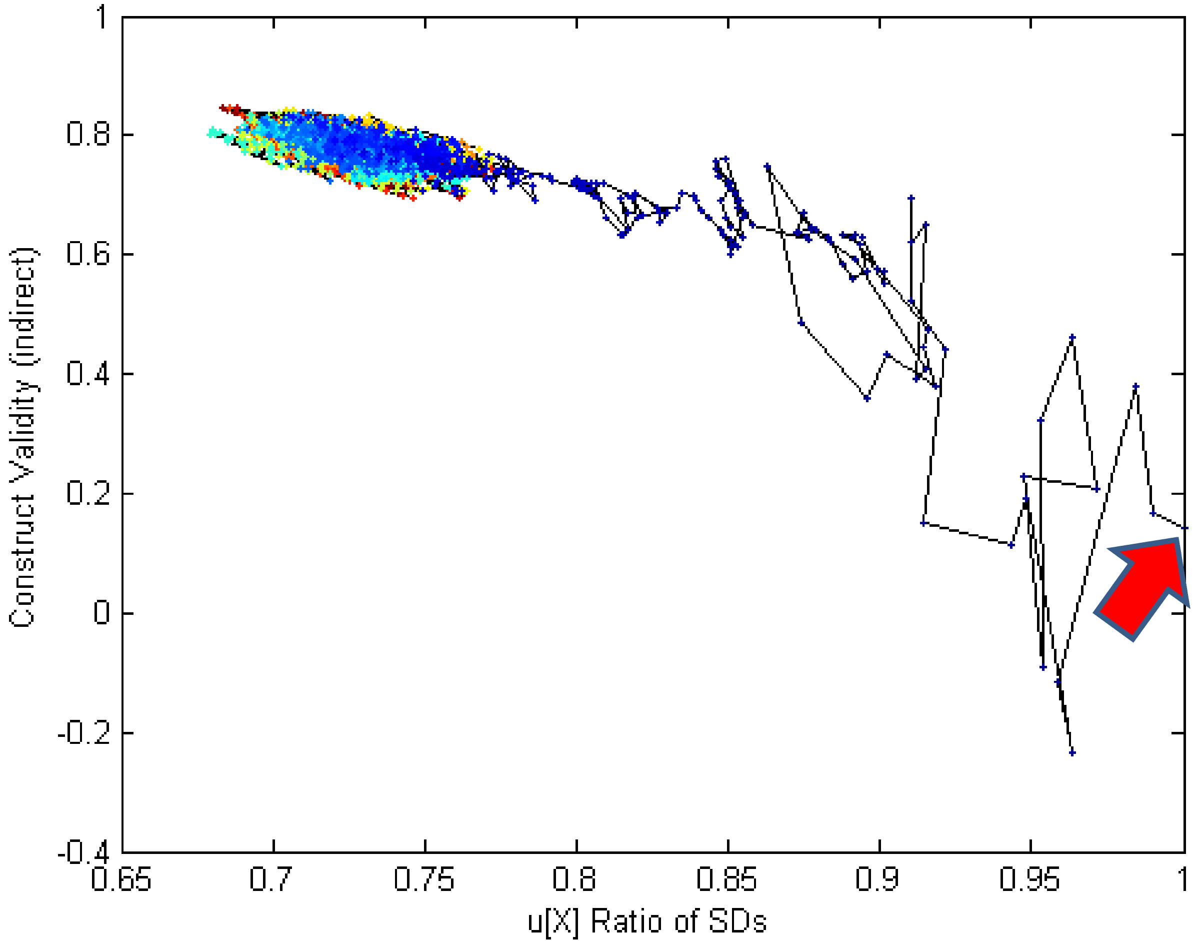

Supplement: Additional file 1 — Statistical appendix: a) Using the MCMC method to extend the Hunter-Schmidt-Le method to include censoring and provide standard errors; and b) The estimation of reliabilities for various measures used in selection studies. [file 1741-7015-11-243-S1.pdf]
